# Behavioral analysis of kainate receptor KO mice and the role of GluK3 subunit in anxiety

**DOI:** 10.1038/s41598-024-55063-z

**Published:** 2024-02-24

**Authors:** Izumi Iida, Kohtarou Konno, Rie Natsume, Manabu Abe, Masahiko Watanabe, Kenji Sakimura, Miho Terunuma

**Affiliations:** 1 Division of Oral Biochemistry, Faculty of Dentistry & Graduate School of Medical and Dental Sciences, Niigata University, Niigata, 951-8514 Japan; 2 Research Center for Advanced Oral Science, Faculty of Dentistry & Graduate School of Medical and Dental Sciences, Niigata University, Niigata, 951-8514 Japan; 3 Department of Anatomy, Faculty of Medicine, Hokkaido University, Sapporo, 060-8638 Japan; 4 Department of Animal Model Development, Brain Research Institute, Niigata University, Niigata, 951-8585 Japan; 5 Department of Life Sciences, Graduate School of Arts and Sciences, The University of Tokyo, Meguro, Tokyo, 153-8902 Japan

**Keywords:** Neuroscience, Ion channels

## Abstract

Kainate receptors (KARs) are one of the ionotropic glutamate receptors in the central nervous system (CNS) comprised of five subunits, GluK1-GluK5. There is a growing interest in the association between KARs and psychiatric disorders, and there have been several studies investigating the behavioral phenotypes of KAR deficient mice, however, the difference in the genetic background has been found to affect phenotype in multiple mouse models of human diseases. Here, we examined GluK1-5 single KO mice in a pure C57BL/6N background and identified that GluK3 KO mice specifically express anxiolytic-like behavior with an alteration in dopamine D2 receptor (D2R)-induced anxiety, and reduced D2R expression in the striatum. Biochemical studies in the mouse cortex confirmed that GluK3 subunits do not assemble with GluK4 and GluK5 subunits, that can be activated by lower concentration of agonists. Overall, we found that GluK3-containing KARs function to express anxiety, which may represent promising anti-anxiety medication targets.

## Introduction

The majority of excitatory neurotransmission in the vertebrate brain is mediated by the ionotropic glutamate receptors (iGluRs)^1^. iGluR family is consisting of four receptors: AMPA, NMDA, kainate, and delta. AMPA receptors (AMPARs) mediate fast excitatory synaptic transmission in the brain and NMDA receptors (NMDARs) regulate synaptic plasticity that underlies learning and memory^2,3^. The delta-type iGluRs do not function as conventional glutamate-gated ion channels^4,5^, but regulate synaptic development and plasticity through their selective binding with the presynaptic neurexin via Cbln1^6,7^.

KARs are widely expressed in the CNS and regulate neurotransmission through diverse mechanisms including postsynaptic depolarization by slow-channel kinetics at a subset of excitatory synapses and presynaptic modulation of glutamate and GABA release^8–10^. The synaptic current of KARs is smaller than that mediated by AMPAR activation; about 10% of total peak current^11,12^. KARs are comprised of five members of subunits, GluK1-GluK5, which show significant differences in the spatio-temporal expression pattern in the brain^13–18^. Different from other iGluRs, KAR subunits can be grouped into low-affinity subunits GluK1-GluK3 and high-affinity subunits GluK4 and GluK5, based on their relative affinity to kainate^13,14,19,20^. In addition, low-affinity GluK1-GluK3 subunits form functional homomeric KARs^19,21–23^, whereas high-affinity GluK4 and GluK5 subunits cannot form homomeric KARs and require heterodimerization with any one of the low-affinity subunits to generate functional KARs^14,24^. Among the various heteromeric subunit combinations, GluK2/GluK5 KARs are known to be the most abundant KARs in the brain^16,25^, and GluK2 and GluK5 have shown to mediate excitatory postsynaptic currents (EPSCs) at mossy fiber synapses in CA3 pyramidal cells^26^. However, studies using high-affinity GluK4 and GluK5 double knockout (KO) mice revealed that the KAR-mediated EPSCs are lost at mossy fiber-CA3 synapses, even though low-affinity subunits are still expressed^27^. Therefore, KARs composed solely of low-affinity subunits have been thought to be activated only under specific conditions such as fast transients of glutamate concentration^28^.

We have previously observed that low-affinity subunits GluK2 and GluK3 are highly expressed in the mouse hippocampus and cerebellum^29^. Using the analytical biochemical approaches, we found that the expression of GluK2 and GluK3 subunits is greater than that of the high-affinity subunits GluK4 and GluK5, indicating possible unknown function of low-affinity homomeric or heteromeric KARs. KARs has been shown to involve in brain diseases such as epilepsy, ischemic brain injury, pain and several mental disorders^30^. Among them, low-affinity KAR genes *GRIK1*, *GRIK2*, and *GRIK3* are reported as one of the candidates for schizophrenia, mania, mild mental retardation, autism and major depression in the human genetic studies^31,32^. Furthermore, GluK1 KO mice has been shown to display anxiety-like behaviors^33,34^, while GluK2 KO mice have been observed to exhibit anxiolytic phenotypes with aggressive behaviors^35^, depressive-like behavior^36^, and reduced social interaction^37^. Altered spatial learning and memory has also been reported in GluK2 KO mice^37^. On the other hand, no behavioral studies have previously been reported in GluK3 KO mice, but the comparative studies of short-term plasticity in wild-type (WT) and GluK2 KO or GluK3 KO mice have identified that GluK2/GluK3 low-affinity heteromers are part of the presynaptic KARs which facilitate hippocampal mossy fiber synaptic transmission^38,39^. In spite of all these evidences suggesting the importance of low-affinity KAR subunits in the CNS, the subunit composition of GluK3-containing KARs and their function in behaviors related to psychiatric disorders remain to be elucidated. Moreover, the behavioral comparison of GluK1-5 single KO mice has never been conducted in the same genetic background of mice.

Here we used all five KAR subunit single KO mice generated in a pure C57BL/6N genetic background and investigated the role of these subunits in animal behavior. By taking advantage of a specific GluK3 antibody that we have produced, and having all GluK1-5 subunit KO mice, we show that GluK3-containing KARs regulate anxiety behavior. Additionally, we characterized the distribution of GluK3 subunit protein in mouse brain and the composition of GluK3-containing receptors, which becomes fundamental information for this subunit. These findings provide important insights into the mechanisms of anxiety-related disorders, which could contribute to the development of therapeutic interventions for such disorders.

## Materials and methods

### Animals

All GluK1-GluK5 KO mice were generated using the ES cell line RENKA derived from the C57BL/6N strain^40^. Detailed characterizations of GluK2-GluK5 KO mice were performed as reported previously^29,41^. The mice were housed in a standardized animal room (lights on 8 a.m. to 8 p.m.; room temperature 22 ± 2 °C), with free access to food and water. The experimental protocols used throughout the study were approved by the Institutional Animal Care and Use Committee of the Niigata University (SA00466) and were performed in accordance with the Japanese regulations on animal experiments and ARRIVE guidelines. Behavioral tests were carried out in 8- to 15-week-old male WT, GluK1 KO (*Grik1*^−/−^), GluK2 KO (*Grik2*^−/−^), GluK3 KO (*Grik3*^−/−^), GluK4 KO (*Grik4*^−/−^), and GluK5 KO (*Grik5*^−/−^) littermates generated by heterozygous breeding and were performed during the light phase (between 10 a.m. and 5 p.m.). Mice were handled for 3 days (3 min per day) before conducting the behavioral tests. After each trial, the apparatus was cleaned with hypochlorous water to prevent a bias due to olfactory cues.

### Open-field test

**Figure 1:**
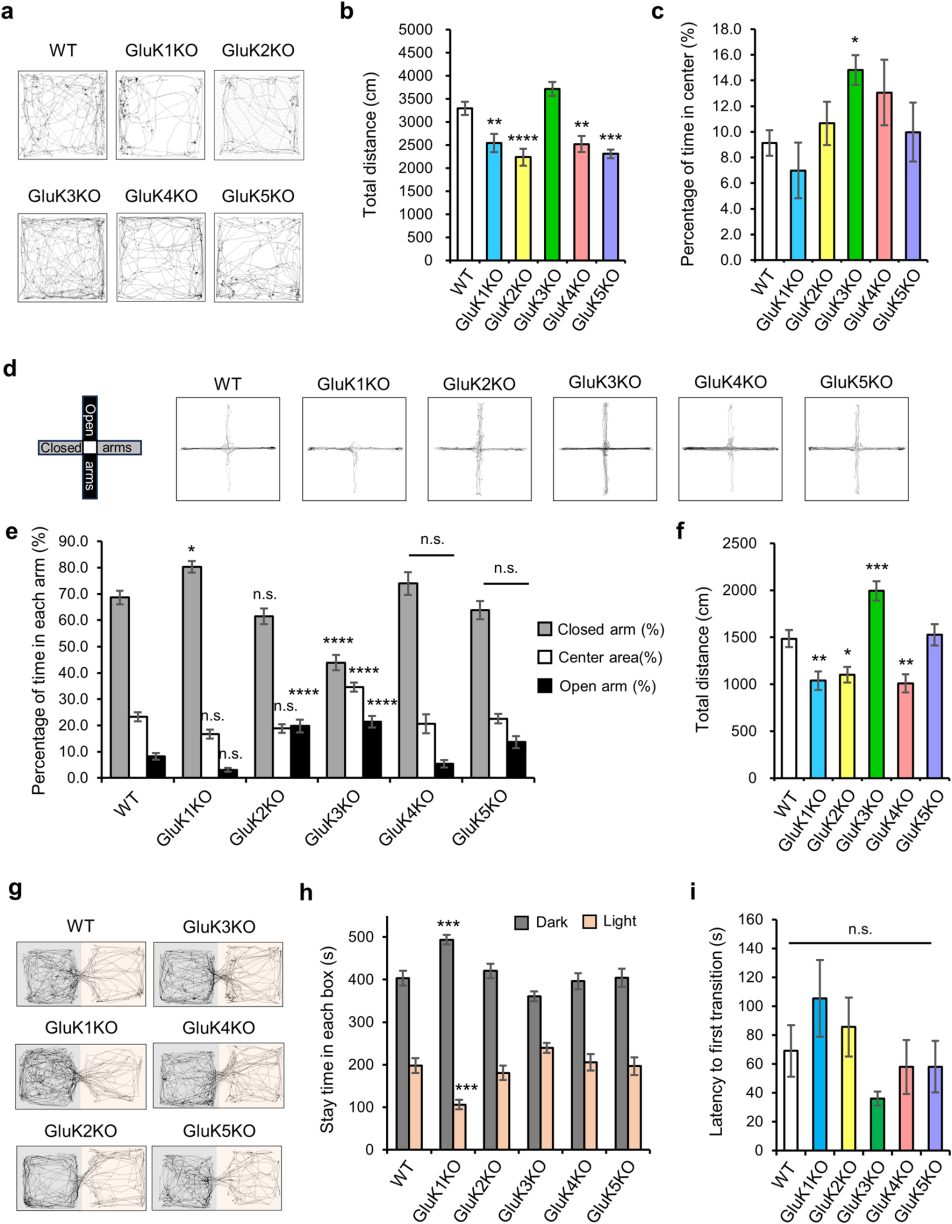
Behavioral analysis of five GluK subunit KO mice. (**a**) Representative trace showing the movement of WT and GluK KO mice in the open field for 10 min (WT: n = 30; GluK1 KO: n = 17; GluK2 KO: n = 21; GluK3 KO: n = 21; GluK4 KO: n = 16; GluK5 KO: n = 17). (**b**) Total walking distance. Reduced total distance traveled was seen in GluK KO mice except for GluK3 KO mice. (**c**) Percentage of time in the central area. Only GluK3KO mice showed increased time spent in the center. (**d**) Representative traces of WT and GluK KO mice in the elevated plus maze test for 10 min. (**e**) Measurement of anxiety with an elevated plus maze test. Altered anxiety-related behavior was identified in GluK1 KO, GluK2 KO and GluK3 KO mice (WT: n = 30; GluK1 KO: n = 20; GluK2 KO: n = 17; GluK3 KO: n = 24; GluK4 KO: n = 16, GluK5 KO: n = 16). (**f**) Total walking distance. Reduced total distance traveled was seen in GluK1, GluK2 and GluK4 KO mice and increased total distance traveled was seen in GluK3 KO mice. (**g**) Representative traces showing the movement of WT and GluK KO mice in the light–dark transition test in 10 min (Gray box: dark chamber, Orange box: light chamber). (**h**) Stay time (s) in each box. Altered anxiety was observed in GluK1 KO mice (WT: n = 26; GluK1 KO: n = 20; GluK2 KO: n = 16; GluK3 KO: n = 24; GluK4 KO: n = 16, GluK5 KO: n = 15). (**i**) Latency to first transition (s) from the dark chamber to the light chamber. No significant difference was observed in GluK KO mice (WT: n = 26; GluK1 KO: n = 20; GluK2 KO: n = 16; GluK3 KO: n = 24; GluK4 KO: n = 16, GluK5 KO: n = 15). Data are mean ± SEM. ns, not significant; *p < 0.05, **p < 0.01, ***p < 0.001, ****p < 0.0001 versus WT mice: one-way ANOVA with Dunnett’s multiple comparisons test.

**Figure 2:**
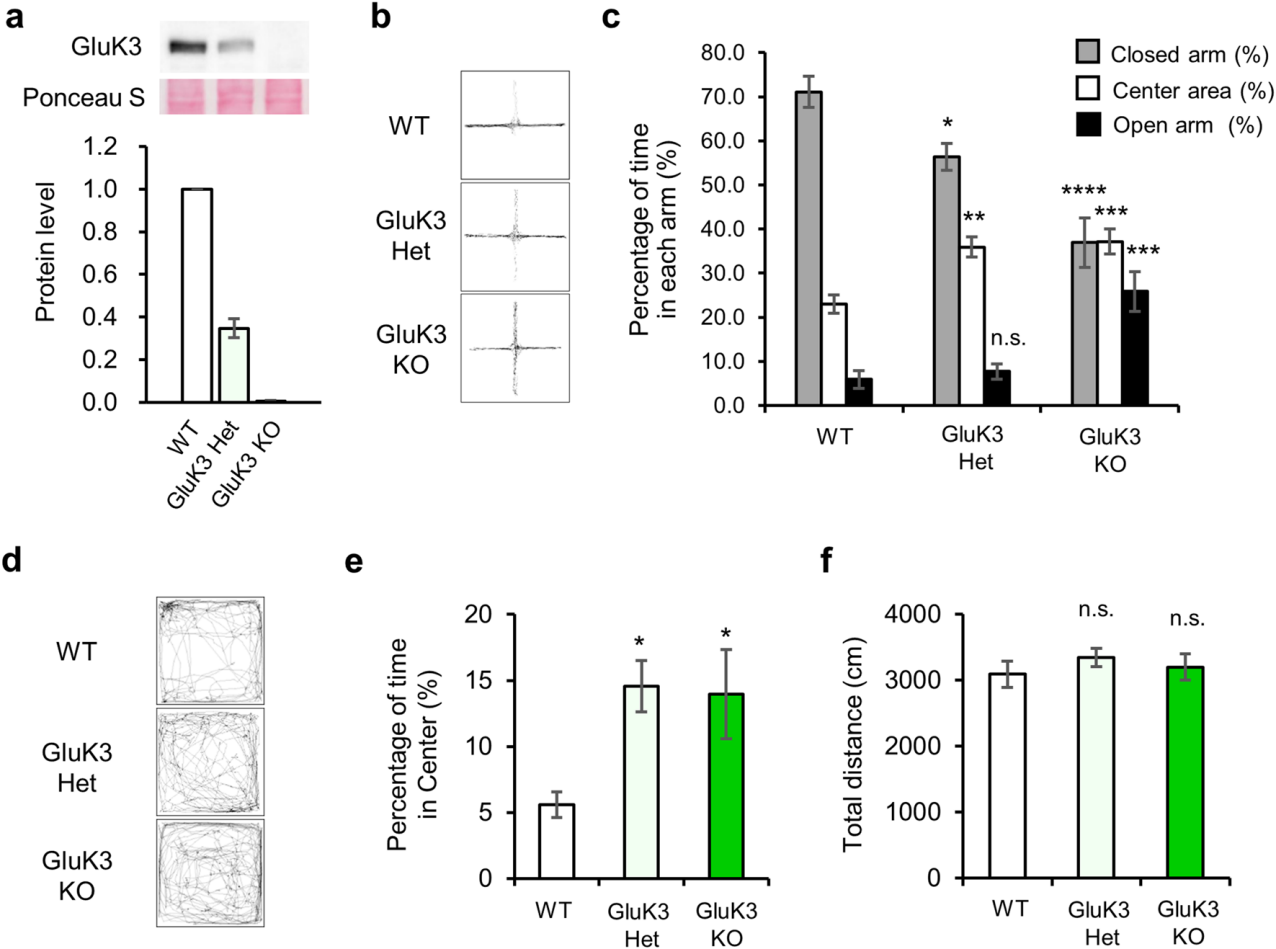
The effect of GluK3 subunit expression levels on anxiety-like behavior. (**a**) The gene dosage reflects the expression levels of GluK3 as detected by western blot analysis using lysates prepared from the cortex of WT, GluK3 Het, and GluK3 KO mice (n = 4). Ablation of GluK3 protein was confirmed in GluK3 KO mice. GluK3 expression was normalized using Ponceau S staining. (**b**) Representative traces showing the movement of WT, GluK3 Het and GluK3 KO mice in the elevated plus-maze test (WT: n = 10; GluK3 Het: n = 9; GluK3 KO: n = 9). (**c**) Percentage of time that WT, GluK3 Het and GluK3 KO mice spent in each arm. Anxiolytic-like behavior was identified in GluK3 Het and KO mice. (**d**) Representative traces showing the spontaneous activities of WT, GluK3 Het, GluK3 KO mice in the open field test. (**e**) Percentage of time spent in the central area of the open field (WT: n = 10; GluK3 Het: n = 8; GluK3 KO: n = 9). Anxiolytic-like behavior was identified in GluK3 Het and KO mice. (**f**) Total walking distance. Total walking distance of GluK3 Het and KO mice were not significantly changed during the open field test. one-way ANOVA with Dunnett’s multiple comparisons test. Data are mean ± SEM. ns, not significant; *p < 0.05, **p < 0.01, ***p < 0.001, ****p < 0.0001 versus WT mice: one-way ANOVA with Dunnett’s multiple comparisons test.

The open field test was carried out using a method described previously^42^. Mice were placed into the corner of an open field apparatus (50 × 50 × 40 cm) (O’Hara & Co. Tokyo, Japan) and allowed to freely explore for 10 min with a chamber illuminated at either 5 lx (Fig.  2d–f) or 100 lx (Figs. 1a–c, S1a–d). During this period, the total distance, the number of moving episodes, moving speed, distance per movement, locomotion frequency, and the time spent in the central region (25% of the total arena) were recorded and automatically calculated by using Image OFCR software (O’Hara & Co., Tokyo Japan; see “Image analysis for behavioral tests”).

### Elevated plus maze test

The apparatus consisted of two open arms (25 × 5 cm) and two enclosed arms of the same size with transparent walls (height 15 cm). The arms and central square area (5 × 5 cm) were made of white plastic plates and elevated 60 cm above the floor (O’Hara & Co., Tokyo, Japan). The same type of arms was oriented opposite from each other. Each mouse was placed in the central square of the maze by facing one of the closed arms. The ratio of the time spent in each area was observed for 10 min under the two different illumination conditions (5 lx; Fig. 2b,c and 100 lx; Figs. 1d–f, S1e). Data acquisition was performed automatically using Image EP software (O’Hara & Co., Tokyo, Japan; see “Image analysis for behavioral tests”).

### Light–dark transition test

The light–dark transition test was carried out using a method described previously^36^. A cage with the size of 21 × 42 × 25 cm was divided into two equal chambers by a black partition containing a small opening (5 × 3 cm height) (O’Hara & Co., Tokyo, Japan). One chamber which is made of a white plastic was brightly illuminated at 525 lx (light chamber), whereas the other chamber was made of a black plastic and not illuminated (dark chamber). Mice were placed in the dark chamber at the beginning of the test and allowed to move freely between the two chambers for 10 min. The time spent in each chamber, latency to the first transition from the dark to light chambers, transition number, and total distance traveled were recorded automatically using Image LD software (O’Hara & Co., Tokyo, Japan; see “Image analysis for behavioral tests”).

### Three-chamber social behavior test

**Figure 3:**
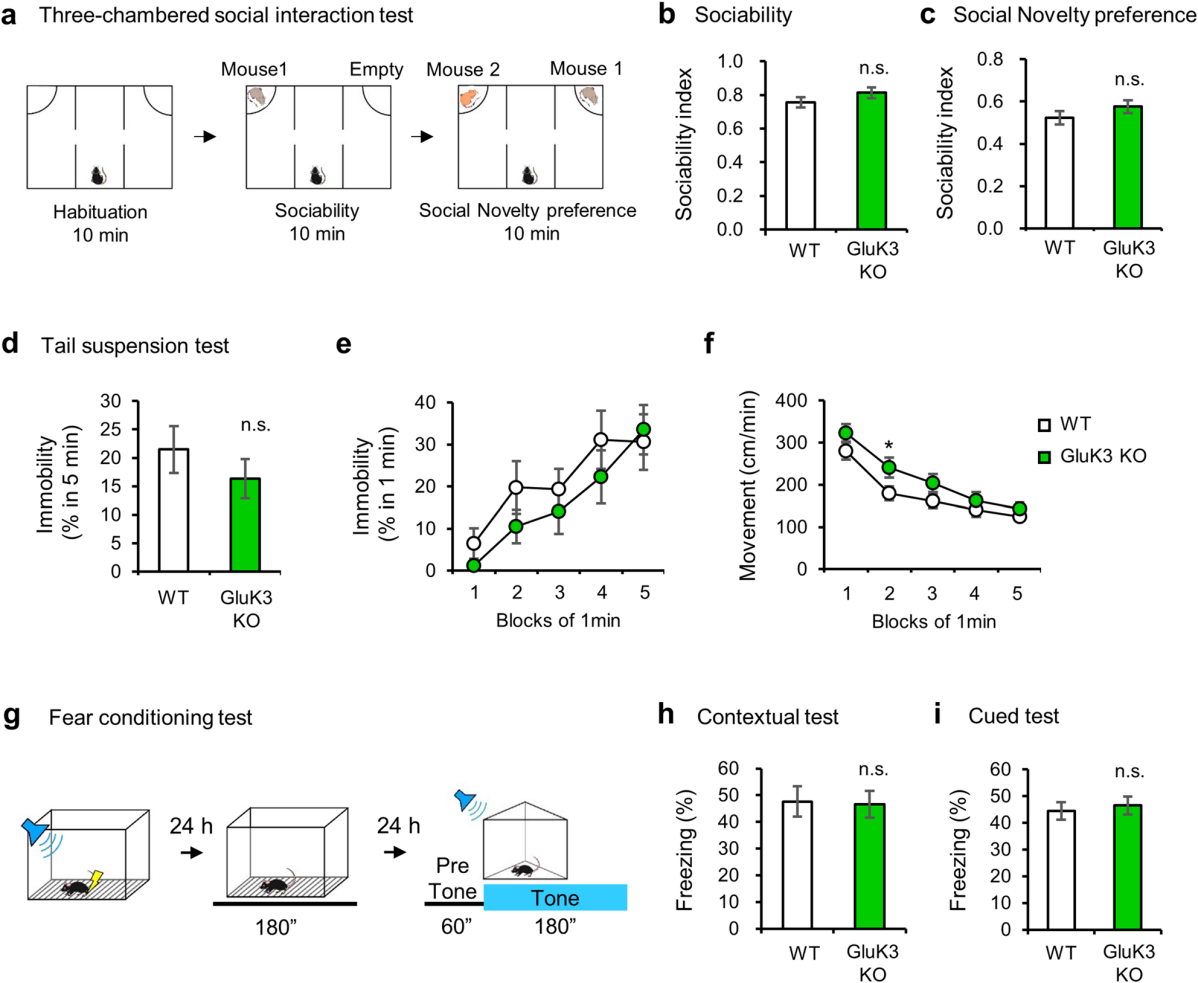
Social skills, depressive-like behaviors and memory performance in GluK3 KO mice. (**a**) Schematic representation of the three-chambered social interaction test (WT, n = 23; GluK3 KO, n = 17). (**b**) Sociability test. No difference in the preference to stay around the cage containing mouse (mouse 1) was observed between WT and GluK3 KO mice (sociability index; *t*(36) = 1.35,* p* = 0.1855). (**c**) Social novelty preference test. No difference in the preference to stay around the cage containing novel mouse (mouse 2) was observed between WT and GluK3 KO mice (social novelty index; *t*(36) = 0.9783,* p* = 0.3345). (**d**) Tail suspension test. The percentage of total immobility time of GluK3 KO mice was not significantly changed compared to WT mice (WT: n = 15; GluK3 KO: n = 20; *t*(33) = 0.9428, *p* = 0.3526). (**e**) Percentage of immobility time from 1 to 5 min. GluK3 KO mice was not significantly changed compared to WT mice. (**f**) Moving distance of mice from 1 to 5 min. GluK3 KO mice moved significantly more than WT mice at 2 min. (**g**) Schematic representation of fear conditioning test (WT: n = 20; GluK3 KO: n = 21). (**h**,**i**) Quantification of contextual freezing and tone-evoked freezing in WT and GluK3 KO mice. No difference in the freezing percentage was observed between WT and GluK3 KO mice on the contextual test 24 h after conditioning (*t*(39) = 0.1347, *p* = 0.8938, (**h**). No difference in the freezing percentage by tone was observed between WT and GluK3 KO mice in cued test 48 h after conditioning (*t*(39) = 0.4429,* p* = 0.6603, (**i**). Data are mean ± SEM. ns, not significant; *p < 0.05, **p < 0.01, ***p < 0.001, ****p < 0.0001 versus WT mice: unpaired *t*-test.

The three-chamber social interaction test was performed as previously described, with minor modification^43^. The apparatus was comprised of a rectangular box consisting of three chambers (O’Hara & Co., Tokyo, Japan). Each chamber measured 20 × 40 × 22 cm, and the dividing walls had small openings (5 × 3 cm) to allow free access to each chamber. Data acquisition and analysis were performed automatically using Image CSI (see “Image analysis for behavioral tests”). Before the test, the mouse was placed in the middle chamber and allowed free exploration through the entire apparatus for 10 min. In the sociability test, a stranger mouse (Mouse 1) (C3H strain; male; purchased from Charles River Laboratories, Yokohama, Japan) having no previous contact with the tested mice was placed in a wire cage on the side of the chambers. The test mouse was placed in the middle chamber and allowed to explore all three chambers freely for 10 min. After that, the subject mouse performed a social novelty preference test for 10 min. The wire cage enclosing Mouse 1 was moved to the opposite side of the chamber that had been emptied during the sociability trial. The unfamiliar C3H male mouse (Mouse 2) was placed in a wire cage on the other side of the chamber. The subject mouse was free to explore both the familiar Mouse 1 and the novel Mouse 2. The number of entering and the time at social zone (6 cm range around social cage) was counted using Image CSI software (O’Hara & Co., Tokyo, Japan; see “Image analysis for behavioral tests”). In Fig. 3, sociability index was calculated as; stay time around Mouse 1 mouse (sec.) divided by stay time around (Mouse 1 (sec.) + Empty (sec.)), and social novelty preference index was calculated as; stay time around stranger Mouse 2 (sec.) divided by stay time around (Mouse 1 mouse (sec.) + Mouse 2 (sec.)). The sociability index and social novelty preference index were also calculated using the number of entries (Fig. S2). The mice which did not stay at the social zone on any one of the cages during the session were removed from the data set (Figs. 3b, S2a,c,e; WT: n = 0, GluK3 KO: n = 1, Figs. 3c, S2b,d,f; WT: n = 2, GluK3 KO: n = 0).

### Tail suspension test

The tail suspension test was performed using a method described previously^36^. Each mouse was suspended 30 cm above the floor by the tail in a chamber made of a white plastic bar (31 × 41 × 41 cm; O’Hara & Co., Tokyo, Japan). The behavior was recorded for 5 min and the immobility and moving distance were measured using Image TS software according to a certain threshold (see “Image analysis for behavioral tests” section). The immobility lasting for less than 2 s was not included in the analysis.

### Fear conditioning test

The fear conditioning test was performed using a method described previously^36^. Each mouse was placed in a transparent acrylic chamber (33 × 25 × 28 cm) with a stainless-steel grid floor (0.2 cm diameter, spaced 0.5 cm apart; O’Hara & Co., Tokyo, Japan) and allowed to explore freely for 3 min. Subsequently, a 65-dB white noise, which served as the conditioned stimulus (CS), was presented for 20 s. During the last 2 s of CS presentation, a foot shock (0.2 mA, 2 s), which served as the unconditioned stimulus (US), was presented. Two more CS-US pairings were presented with an inter-stimulus interval of 40 s. Twenty-four hours after the conditioning, a contextual test was conducted in the same chamber. Forty-eight hours after the conditioning, a cued fear memory was tested in a triangular chamber (33 × 33 × 32 cm) made of an opaque white plastic and the mice were allowed to explore freely for 1 min. Subsequently, each mouse was given CS presentation for 3 min. In each session, the percentage of freezing was calculated automatically using Image FZ software (O’Hara & Co., Tokyo, Japan; see “Image analysis for behavioral tests”).

### Image analysis for behavioral tests

Analysis for behavioral tests was performed using a method described previously^36^. The application software used for the behavioral studies (Image OFCR, EP, LD, CSI, TS, and FZ) was based on the public domain NIH Image program developed by the U.S. National Institutes of Health (available at http://rsb.info.nih.gov/nih-image/) and ImageJ program (http://rsb.info.nih.gov/ij/), which were modified for each test (available through O’Hara & Co., Tokyo, Japan).

### Antibody

Anti-GluK3 antibody raised in rabbit against C-terminal 17 amino acid residues of mouse GluK3 (903–919, NM_001081097) was used for immunohistochemistry, immunoprecipitation, and western blotting. The rabbit polyclonal antibodies anti-GluK2 (Synaptic Systems, 180 003), anti-GluK4^41^, and anti-GluK5 (Millipore, 06-315) were used for immunoprecipitation and western blotting. The specificities of these antibodies have been determined previously^29^. Anti-GluN1 (BD Biosciences, 556308), anti-GluN2A (BD Biosciences, 612286), anti-GluN2B (BD Biosciences, 610416), anti-GluA1 (Frontier Institute, Japan, MSFR102270), anti-GluA2 (Millipore, MAB397), anti-GluA3^44^, anti-PSD-95 (Santa Cruz Biotechnology, SC32290), anti-D1R (Frontier Institute, Japan, MSFR101030), and anti-D2R (Frontier Institute, Japan, MSFR101060) were used for western blotting.

### Fixation and the preparation of histological sections

Brain fixation was performed using a method described previously^36^. Mouse brains were freshly obtained under deep pentobarbital anesthesia (Maruishi Pharmaceutical, Japan) and immediately frozen in powdered dry ice for the preparation of fresh frozen sections (20 μm). Fresh frozen sections were air-dried and fixed by dipping in 4% paraformaldehyde in 0.1 M phosphate buffer (pH 7.2) for 15 min before immunohistochemistry.

### Immunohistochemistry

All immunohistochemical incubations were performed at room temperature. The sections were incubated with 10% normal donkey serum or goat serum for 20 min, followed by an incubation with polyclonal rabbit anti-GluK3 antibody (1 μg/mL) overnight. The sections were then incubated with Alexa Fluor 488 goat anti-rabbit IgG antibody for 2 h at a dilution of 1:1000 (Thermo Fisher Scientific, A-11008). Images were taken with a confocal laser-scanning microscope (Zeiss LSM710; Carl Zeiss, Germany and BZ-X800; Keyence, Japan).

### Preparation of the brain samples

Each brain region of adult WT, GluK3 Het and GluK3KO mice at 8–12 weeks old were immediately dissected after euthanasia. The striatum tissues of D1R KO and D2R KO mice were gifts from Prof. T. Sasaoka at Niigata University, Niigata, Japan^45^. Brain tissues were homogenized in 0.1% sodium dodecyl sulfate (SDS) plus Buffer A (20 mM Tris–HCl (pH 8.0), 150 mM NaCl, 5 mM EDTA, 10 mM NaF, 2 mM Na_3_VO_4_, 10 mM Na_4_P_2_O_7_, 1% Triton X-100, 10 mg/mL leupeptin, 1 mg/mL pepstatin A and 1 mg/mL antipain) as previously described^46^. The lysates from each brain sample were centrifuged at 12,200×*g* for 30 min at 4 °C, and the supernatants were used for western blotting.

### Western blotting

The standard western blot protocol was used as described previously^47^. Protein samples were separated by SDS–polyacrylamide gel electrophoresis and transferred to a supported nitrocellulose membrane (Cytiva, 10600001). The membrane was stained with Ponceau S (Sigma-Aldrich, P3504) for protein normalization, blocked with a blocking buffer (5% non-fat milk in Tris Buffered Saline with Tween 20) for 1 h and probed with primary antibodies. Membranes were then probed with horseradish peroxidase-conjugated secondary antibodies (GE Healthcare, NA934, NA931) and visualized by an enhanced chemiluminescent substrate (SuperSignal West Dura Extended Duration Substrate, Thermo Fisher Scientific). Blots were quantified using the CCD-based Amersham Imager 680 system (Cytiva) and the intensity of bands was measured using NIH ImageJ. The intensity of each band was normalized by Ponceau S staining and divided by the intensity of WT band.

### Immunoprecipitation

The GluK2, GluK3, GluK4, and GluK5 antibodies (1 μg) were bound to 40 μL of Protein A agarose beads (Sigma, P1406) at 4 °C for over 1 h. The mouse cortex was lysed in Buffer A and centrifuged at 12,200×*g* for 30 min at 4 °C. The resulting supernatant was incubated with the antibody-conjugated Protein A agarose beads in Buffer A at 4 °C overnight. The beads were precipitated by centrifugation and washed twice with Buffer A plus 500 mM NaCl and four times with Buffer A, followed by an SDS-PAGE and western blotting with GluK2-5 antibodies.

### Anti-psychotropic drug administration

Risperidone was intraperitoneally injected 30 min prior to the behavioral tests and haloperidol was intraperitoneally injected 1 h before the tests. The dosages were 0.04 mg/kg for risperidone (Tokyo Chemical Industry, R0087) and 0.1 mg/kg for haloperidol (Wako, 080-04263). All drugs were suspended in a minimal volume of  DMSO and dissolved in 0.9% saline to a final concentration of 0.1% for risperidone and 0.04% for haloperidol.

### Experimental design and statistical analysis

Statistical analyses were conducted with GraphPad Prism7 (GraphPad Software Inc.). For the behavioral analysis of Figs. 1 and 2 and comparison of protein expression levels in Fig. 2a, data were analyzed using one-way ANOVA with Dunnett’s post-hoc test between WT and each genotype. The behavioral analysis of Figs. 3, 6 and 7 were done by two-tailed unpaired *t*-test. Behavioral tests were performed by investigators with knowledge of the identities of the experimental groups. All behavioral experiments were controlled by the computer systems, with data collected and analyzed in an automated and unbiased way. Comparisons of protein expression of glutamate receptor subunits between WT and each genotype were performed using one-way ANOVA followed by Dunnett’s post-hoc test comparisons. The protein expression of D1R and D2R between WT and GluK3 KO mice were compared by Mann–Whitney U test. Individual sample sizes for the tests (n) are indicated in the figure legends. All data are presented as mean ± SEM. Differences were considered significant at *p* < 0.05.

## Results

### Basal motor activity is reduced in KAR subunit KO mice except for GluK3 KO mice

We generated all five KAR subunit single knockout mice (GluK1, GluK2, GluK3, GluK4 and GluK5 KO mice) in a pure C57BL/6N background, and examined their general motor activity using the open field test (Fig. 1a). We found reduced total distance traveled as well as reduced distance per movement (distance/movement) in GluK1 KO, GluK2 KO, GluK4 KO and GluK5 KO mice compared to that in WT mice from the same ES cell line, and only GluK3 KO mice showed similar motor activity to WT mice (Total distance: *F*(5, 116) = 14.27, *p* < 0.0001; GluK1 KO, *p* = 0.0044; GluK2 KO, *p* = 0.0001; GluK3 KO, *p* = 0.1611; GluK4 KO, *p* = 0.0039; GluK5 KO, *p* = 0.0001; Distance/movement: *F*(5, 116) = 7.703, *p* < 0.0001; GluK1 KO, *p* = 0.0149; GluK2 KO, *p* = 0.0001; GluK3 KO, *p* = 0.9924; GluK4 KO, *p* = 0.028; GluK5 KO, *p* = 0.0018; Fig. 1b and S1a). Furthermore, moving speed was reduced in GluK2 KO, GluK4 KO and GluK5 KO mice compared to that in WT mice (*F*(5, 116) = 9.865, *p* < 0.0001; GluK1 KO, *p* = 0.073; GluK2 KO, *p* = 0.0001; GluK3 KO, *p* = 0.8924; GluK4 KO, *p* = 0.0018; GluK5 KO, *p* = 0.0035; Fig. S1b). Additionally, we examined total distance traveled in littermate control (WT) from each KAR subunit KO mouse lines and found that they display similar locomotor activity level (Fig. S1d). These results suggested that KAR subunits contribute to locomotor activity, but GluK3 may have different roles from the rest of KAR subunits.

### GluK3 KO mice exhibit anxiolytic-like behaviors

Open field test is often used to evaluate anxiety-like behavior in rodents^48^. We found that only GluK3 KO mice exhibited an increased time spent in the center area, suggesting an anxiolytic-like phenotype in these mice (*F*(5, 116) = 2.552, *p* = 0.0314; GluK1 KO, *p* = 0.8219; GluK2 KO, *p* = 0.9356; GluK3 KO, *p* = 0.0464; GluK4 KO, *p* = 0.3398; GluK5 KO, *p* = 0.9961, Fig. 1c). The locomotion frequency of GluK3 KO mice was similar to WT mice (*F*(5, 116) = 6.051, *p* < 0.0001; GluK1 KO, *p* = 0.1409; GluK2 KO, *p* = 0.0252; GluK3 KO, *p* = 0.1826; GluK4 KO, *p* = 0.1867; GluK5 KO, *p* = 0.0675; Fig. S1c). To validate if GluK3 KO mice are altered in anxiety, we performed the elevated plus maze test (Fig. 1d). We analyzed the total distance traveled in each genotype (Fig. 1f) and found slight reduction in the locomotion in GluK1 KO, GluK2 KO and GluK4 KO mice (WT, 1485.35 cm; GluK1 KO, 1038.4 cm, *p* = 0.0043; GluK2 KO, 1102.5 cm, *p* = 0.0282; GluK4 KO, 1011.4 cm, *p* = 0.0049), and increased locomotion in GluK3 KO mice (1992.8 cm, *p* = 0.0004). No difference was observed in GluK5 KO mice (GluK5 KO, 1527.8 cm, *p* = 0.998). We then determined the time staying in each arm and found that compared to WT mice, GluK3 KO mice spent more time in the open arm and center area but less time in the closed arm (Closed arm: *p* = 0.0001; Center area: *p* = 0.0001; Open arm: *p* = 0.0001, Fig. 1e). Additionally, we observed increased time in the closed arm in GluK1 KO mice and increased time in the open arm in GluK2 KO mice compared to WT mice respectively (Closed arm: *F*(5, 117) = 17.85, *p* < 0.0001; GluK1 KO, *p* = 0.00191; GluK2 KO, *p* = 0.3035; Center area: *F*(5, 117) = 10.28, *p* < 0.0001; GluK1 KO, *p* = 0.0616; GluK2 KO, *p* = 0.3758; Open arm: *F*(5, 117) = 17.29, *p* < 0.0001; GluK1 KO, *p* = 0.1383; GluK2 KO, *p* = 0.0001, Fig. 1e). From the measurements of entry into each arm, we found reduced entry into both closed and open arms in GluK1 KO, reduced entry into closed arm in GluK2 KO mice, and increased entry into open arm in GluK3 KO mice (Closed arm: *F*(5, 117) = 10.12, *p* < 0.0001; GluK1 KO, *p* = 0.0002; GluK2 KO, *p* = 0.001; GluK3 KO, *p* = 0.3726; GluK4 KO, *p* = 0.2736; GluK5 KO, *p* = 0.4905; Open arm: *F*(5, 117) = 6.067, *p* < 0.0001; GluK1 KO, *p* = 0.00318; GluK2 KO, *p* = 0.5139; GluK3 KO, *p* = 0.0239; GluK4 KO, *p* = 0.9686; GluK5 KO, *p* = 0.8284, Fig. S1e). Altered total number of entry was observed only in GluK1 KO mice (*F*(5, 117) = 7.743, *p* < 0.0001; GluK1 KO, *p* = 0.0002; GluK2 KO, *p* = 0.0626; GluK3 KO, *p* = 0.1015; GluK4 KO, *p* = 0.3858; GluK5 KO, *p* = 0.9572, Fig. S1e). Together, these results suggested the expression of anxiolytic-like behavior in GluK3 KO mice and possibly in GluK2 KO mice, and anxiety-like behavior in GluK1 KO mice. In addition, we performed the light/dark transition test (Fig. 1g–i), another test that can examine anxiety in rodents. We found no difference in the latency to first transition in any of KAR KO mice compared to WT mice (*F*(5, 111) = 1.92, *p* = 0.0966; GluK1 KO, *p* = 0.3982; GluK2 KO, *p* = 0.9472; GluK3 KO, *p* = 0.4478; GluK4 KO, *p* = 0.9905; GluK5 KO, *p* = 0.9921, Fig. 1i). However, we observed the expression of anxiety in GluK1 KO mice (Dark box: *p* = 0.0002; Light box: *p* = 0.0002; Fig. 1h). On the other hand, we could not detect significant anxiolytic behavior in GluK3 KO mice, although the mice showed trend towards anti-anxiety, which can be determined by reduced time in the dark box and increased time in the light box (Dark box: *p* = 0.1317; Light box: *p* = 0.161; Fig. 1h). Of note, we found significantly increased total distance traveled in GluK3 KO mice and reduced total distance traveled in GluK1 KO and GluK2 KO mice (*F*(5, 111) = 9.636, *p* < 0.0001; GluK1 KO, *p* = 0.0422; GluK2 KO, *p* = 0.0449; GluK3 KO, *p* = 0.0098; GluK4 KO, *p* = 0.9999; GluK5 KO, *p* = 0.0633; Fig. S1g). The transition number was significantly reduced in GluK1 KO, GluK2 KO, and GluK5 KO mice (*F*(5, 111) = 7.568, *p* < 0.0001; GluK1 KO, *p* = 0.0074; GluK2 KO, *p* = 0.0003; GluK3 KO, *p* = 0.9442; GluK4 KO, *p* = 0.9999; GluK5 KO, *p* = 0.033; Fig. S1f).

Since GluK3 KO mice displayed a significant anxiolytic-like behavior in open field test and elevated plus maze test, next we tested if the amount of GluK3 in the CNS influences the expression of this behavior. Before conducting behavioral tests, we confirmed genotype-dependent GluK3 protein levels in GluK3 Het, KO and littermate WT mice using the specific antibody we generated previously^29^ (Fig. 2a). In the elevated plus maze test, we found reduced time in the closed arm as well as increased time in the center areas in GluK3 Het and KO mice than in littermate WT mice (Closed arm: (*F*(2, 25) = 16.99, *p* < 0.0001, GluK3 Het: *p* = 0.0348; GluK3 KO: *p* = 0.0001, Center area: *F*(2, 25) = 11.25, *p* = 0.0003, GluK3 Het: *p* = 0.0013; GluK3 KO: *p* = 0.0005, Fig. 2b,c). We also observed that GluK3 KO mice stay longer in the open arm than the GluK3 Het mice, suggesting more distinct anxiolytic phenotype in GluK3 KO mice (Open arm: *F*(2, 25) = 13.84, *p* < 0.0001, GluK3 Het: *p* = 0.8792; GluK3 KO: *p* = 0.0001, Fig. 2c). In open field test, we identified that both GluK3 Het and KO mice spent significantly longer time in the center area of the open field compared to littermate WT mice (*F*(2, 24) = 5.014, *p* = 0.0152; GluK3 Het, *p* = 0.0206; GluK3 KO, *p* = 0.0259, Fig. 2d,e). Of note, no difference in the total distance traveled was seen between genotypes in the open field test (*F*(2, 24) = 0.4541, *p* = 0.6403, Fig. 2f). Together, these results suggested that GluK3 KO mice are less anxious than the WT mice and there is a correlation between the global level of GluK3 and the anxiety-related behavior.

### Sociability, depression-like behavior, and memory are normal in GluK3 KO mice

To examine whether there were other behavioral characteristics in GluK3 KO mice, we conducted a three-chamber social interaction test to assess sociability (Fig. 3a–c), a tail suspension test to analyze depression-like behavior (Fig. 3d–f), and a fear conditioning test to assess fear-associated memory (Fig. 3g–i). We found that both GluK3 KO and littermate WT mice spend more time around the Mouse 1-containing cage than the empty cage (sociability index; WT: 0.76; GluK3 KO: 0.81, Fig. 3b). Both genotypes also preferred to spend time around the novel Mouse 2-containing cage than the familiar Mouse 1-containing cage (social novelty index; WT: 0.52; GluK3 KO: 0.58, Fig. 3c). In addition, we examined the number of entries around the cages and observed no changes between genotypes in both the sociability test and the social novelty test (sociability index; WT: 0.67; GluK3 KO: 0.71; social novelty index; WT: 0.5; GluK3 KO: 0.6, Fig. S2a,b). Furthermore, we examined the % of time as well as the number of entries in each box during 10 min of sociability test and social novelty preference test, and found no difference between WT and GluK3 KO mice (Fig. S2c–f). All together, these results suggested that GluK3 does not influence the sociability. The GluK3 gene is known as a susceptibility factor of recurrent major depressive disorder^49^. In our tail suspension test, GluK3 KO mice did not show an alteration in the % of immobility time compared to littermate WT mice, although GluK3 KO mice were reduced in their immobility time than the WT mice (Fig. 3d,e). However, from the measurement of moving distance, we found increased moving distance in GluK3 KO mice at 2 min (Fig. 3f), suggesting that anti-depressive-like phenotype is expressed in the mice. Previous studies have reported that some KAR subunit deficient mice demonstrate impaired memory; spatial memory in GluK2 KO^37^ and GluK4 KO mice^50^ and fear memory in GluK2 KO and GluK5 KO mice^36,51^. We performed the contextual and cued fear conditioning test which assess the ability of mice to learn and remember an association between environmental cues and aversive experiences (Fig. 3g). We found no difference between GluK3 KO and littermate WT mice (Fig. 3h,i). Together, these results indicated that GluK3 is dispensable for sociability and contextual memory, but plays role in anxiety-like behavior and to some degree in depression-like behavior.

### GluK3 is expressed in the brain regions associated with anxiety

**Figure 4:**
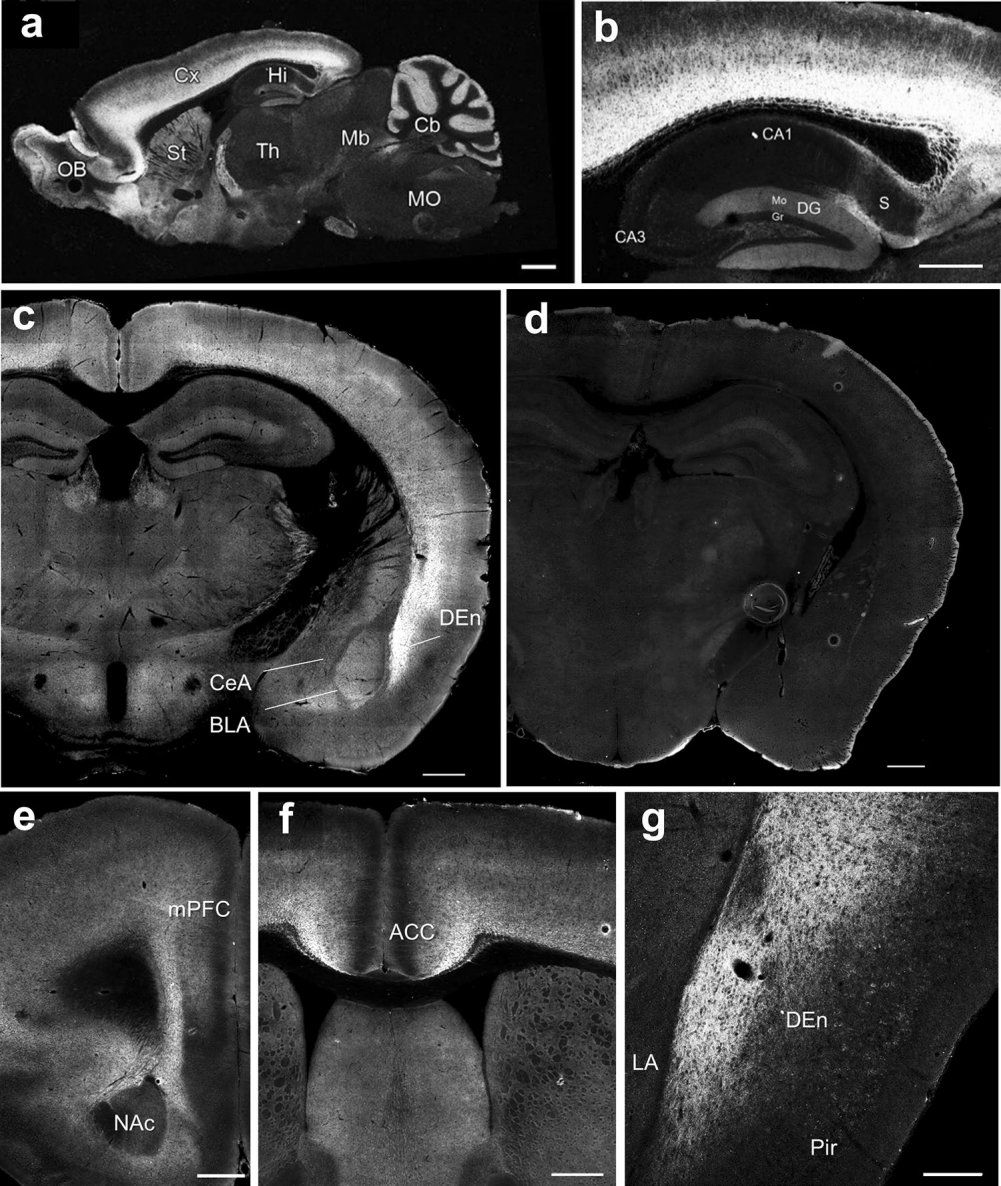
Expression of GluK3 subunit proteins in the mouse brain. (**a**) Immunohistochemical staining of GluK3 in the sagittal section of the adult mouse brain. An intense labeling of GluK3 was found in the olfactory bulb (OB), cortex (Cx), and cerebellum cortex (Cb) and mild labeling was found in the striatum (St) and hippocampus (Hi). (**b**) GluK3 expression in the hippocampus. GluK3 labeling was found in the subiculum (S) and the molecular layer (Mo) of the dentate gyrus (DG). (**c**,**d**) GluK3 expression in the coronal section of the adult WT brain (**c**) and GluK3 KO brain (**d**). Intense labeling was found in the dorsal endopiriform nucleus (DEn) and weak labeling was found in the central nucleus of the amygdala (CeA) and the basolateral amygdala (BLA). (**e**,**f**) Distribution of GluK3 in the cortex. GluK3 was observed in the medial prefrontal cortex (mPFC) and the anterior cingulate cortex (ACC). (**g**) Intense labeling of GluK3 were found in the DEn of the coronal section. Th, thalamus; Mb, midbrain; MO, medulla oblongata; CA1 and CA3, CA1 and CA3 regions of the Ammon’s horn; Gr, granule cell layer. Scale bar represents: (**a**) 1 mm, (**b**–**g**), 500 μm.

To determine the localization of GluK3 in the mouse brain, we performed immunohistochemical analysis with sagittal and coronal sections of WT and GluK3 KO brain using our specific antibody for GuK3 subunit. GluK3 immunoreactivity was widely distributed in the olfactory bulb, cerebral cortex, striatum, and cerebellar cortex at high levels (Fig. 4a). GluK3 subunit expression was also observed in the basolateral amygdala, an important region for emotional processing (Fig. 4c). Although hippocampus is the most studied area of the brain to understand the physiology of KARs, GluK3 expression was moderate in the dentate gyrus and lower in the CA1 and CA3 mossy fiber (Fig. 4b). Intense signal for GluK3 was detected in the medial prefrontal cortex (mPFC) and anterior cingulate cortex (ACC) that are associated with anxiety (Fig. 4e,f). Consistent with the expression pattern of GluK3 mRNA^19^, we found enriched GluK3 signal in the dorsal endopiriform nucleus (DEn) (Fig. 4g). Of note, we confirmed the absence of immunostaining in GluK3 KO brain (Fig. 4d) in addition to western blotting (Fig. 2a).

### GluK3 neither assembles with GluK4 nor GluK5 subunits but forms heteromer with GluK2 subunit in mouse cortex

**Figure 5:**
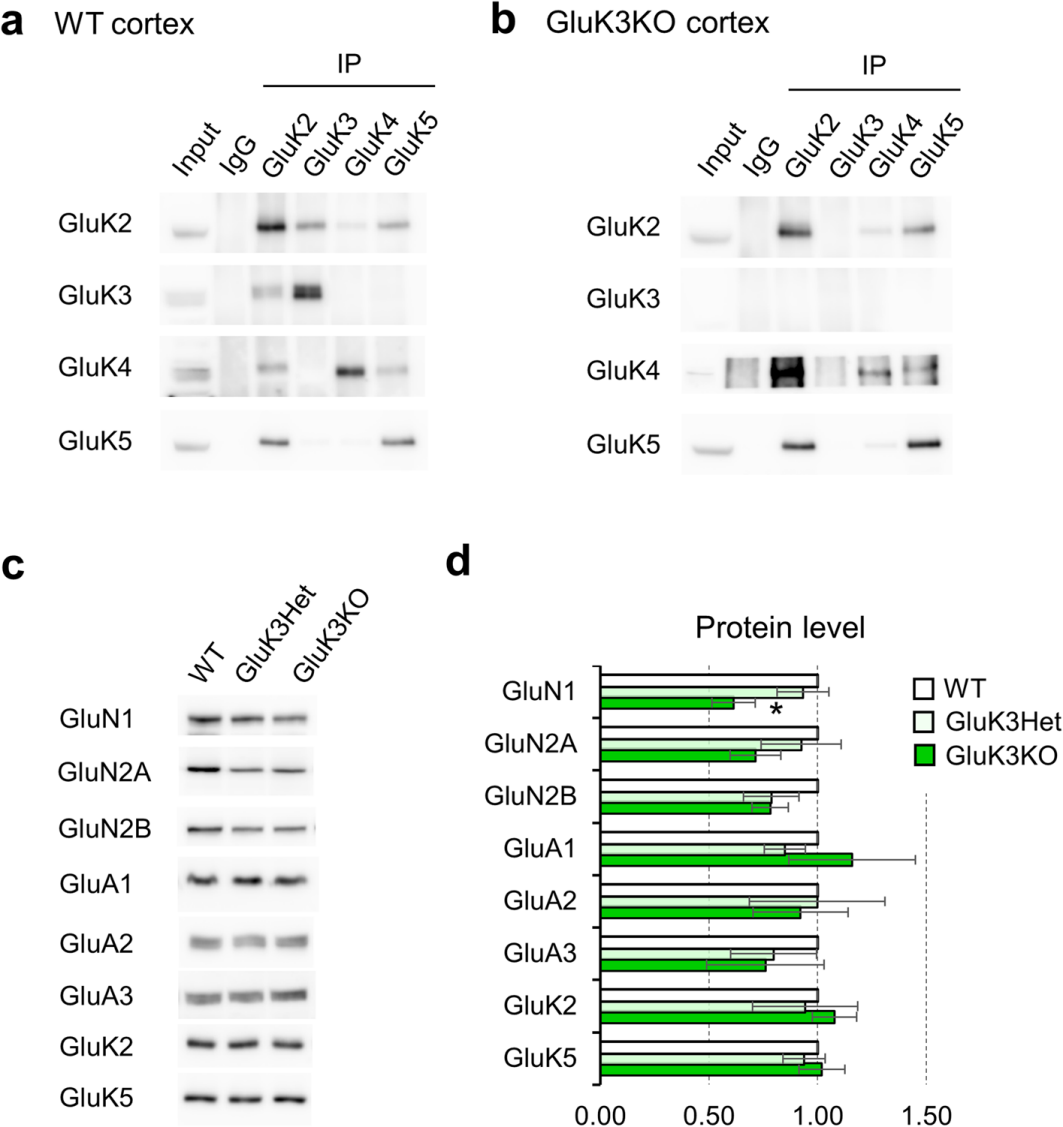
Co-immunoprecipitation of GluK subunits and the effect of GluK3 deficiency on the expression of other iGluRs. (**a**) Immunoprecipitation in the lysate prepared from WT mouse cortex. GluK3 co-precipitates with GluK2 but not with GluK4 and GluK5. n = 3. (**b**) Immunoprecipitation in the lysate prepared from GluK3 KO mouse cortex. GluK2 co-precipitates with GluK4 and GluK5 without GluK3. n = 3. (**c**) Representative blots for iGluR subunits using the cortical lysates prepared from WT, GluK3 Het and GluK3 KO mice. Equal amounts of protein samples (15 μg) were loaded in each lane. (**d**) Quantification of iGluR subunits. Decreased protein levels of GluN1 but not the other subunits were observed in GluK3 KO mice. Each iGluR expression was normalized using Ponceau S staining. Data are mean ± SEM. *p < 0.05 versus WT, one-way ANOVA with Dunnett’s multiple comparisons test.

To identify the subunit composition of GluK3-containing KARs in native mouse brain, we performed co-immunoprecipitation experiments in mouse cortex using the antibodies for low-affinity subunits (GluK2 and GluK3) and the high-affinity subunits (GluK4 and GluK5) that we generated previously^29^ (Fig. 5a). We found that GluK3 only co-precipitated with GluK2, suggesting that GluK3 mostly form low-affinity KARs in the cortex. On the other hands, GluK2 was co-precipitated with GluK3, GluK4, and GluK5. Supporting this data, GluK4 was co-precipitated with GluK2 and GluK5, and GluK5 was co-precipitated with GluK2 and GluK4. Immunoprecipitation in GluK3 KO mice confirmed the lack of GluK2/GluK3 heteromeric KARs in the GluK3 KO brain, however, GluK2/4, GluK2/5 and GluK4/5 KARs were intact (Fig. 5b). The ablation of GluK3 containing low-affinity KARs could affect neurotransmission mediated by other ionotropic glutamate receptors due to altered neuronal activity. To test this possibility, we examined the expression of AMPARs and NMDARs in total lysate prepared from cortex in WT, GluK3 Het and GluK3 KO mouse littermate (Fig. 5c). We observed a decrease in the levels of GluN1 in GluK3 KO mice (*F*(2, 6) = 7.208, *p* = 0.0254, GluK3 Het: *p* = 0.4032; GluK3 KO: *p* = 0.0172), but not in other subunits (Fig. 5d). The expression of GluK2 and GluK5, which are proposed to be the major KAR subunits in the brain^29,52^, was not altered in GluK3 Het or KO mice.

### The dopamine D2 receptor-induced anxiety-like behavior is altered in GluK3 KO mice

**Figure 6:**
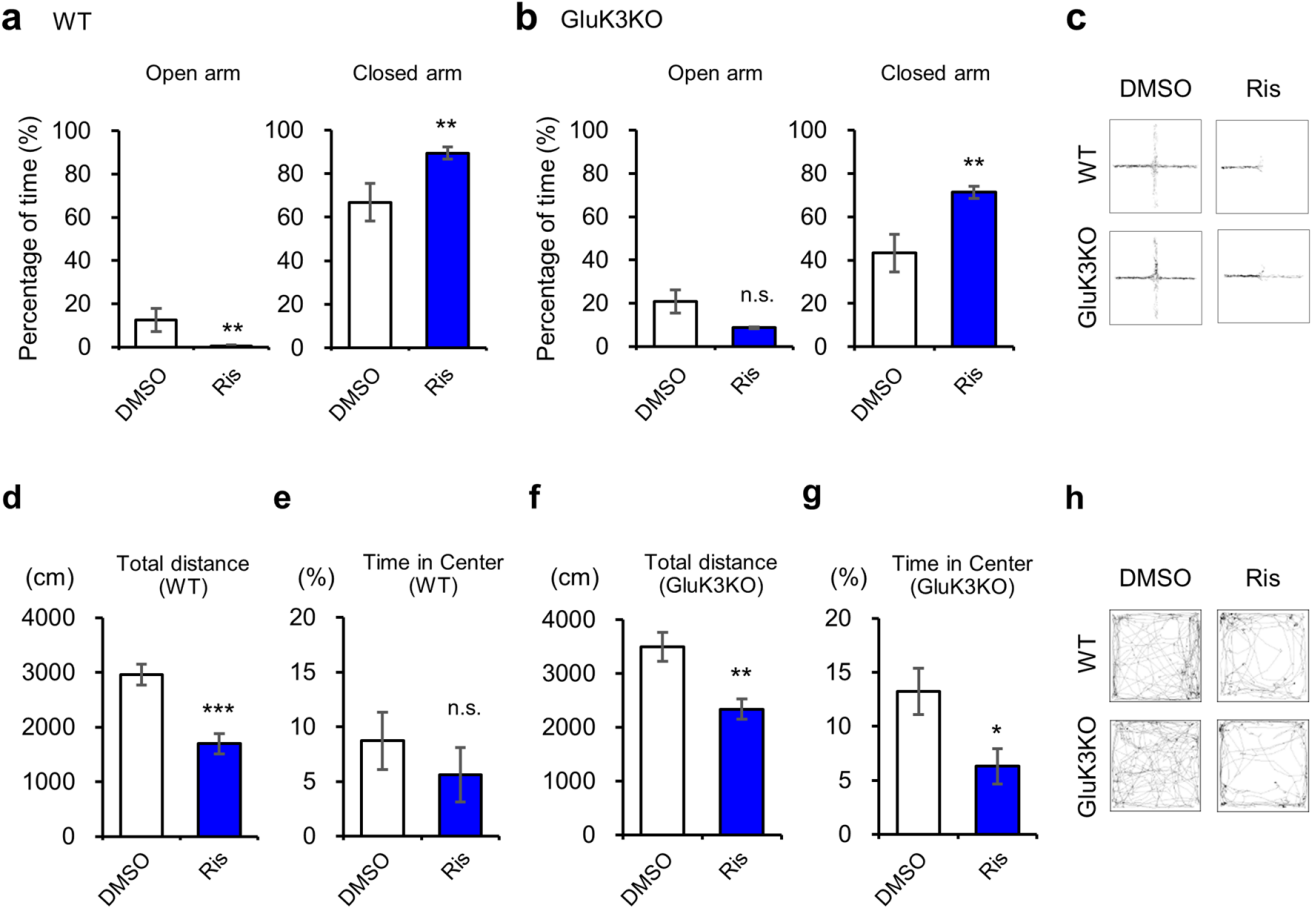
Effects of risperidone on anxiety-related behavior in WT and GluK3 KO mice. (**a**) Percentage of time spent in both open and closed arms in WT mice treated with DMSO or risperidone (Ris) (0.04 mg kg^−1^) during the 10 min of elevated plus maze test (DMSO, n = 8; Ris, n = 14; Open arm: *t*(20) = 3.028, *p* = 0.0066; Closed arm: *t*(20) = 3.045, *p* = 0.0064). (**b**) Percentage of time spent in the open and closed arms in GluK3 KO mice treated with DMSO or risperidone (DMSO, n = 10; Ris, n = 10; Open arm: *t*(18) = 1.866, *p* = 0.0784; Closed arm: *t*(18) = 3.432, *p* = 0.003). (**c**) Representative traces of DMSO or risperidone-treated WT and GluK3 KO mice in the elevated plus maze test. (**d**) Total distance travelled in WT mice in the open field test. WT mice traveled significantly shorter distance during the 10 min of test after risperidone injection. DMSO, n = 10; Ris, n = 14; *t*(22) = 4.609 , *p* = 0.0001. (**e**) Percentage of time spent in the central area of the open field in WT mice. The percentage of time in the center was unaltered in WT mice administrated with risperidone. (**f**) Total distance travelled in GluK3 KO mice in the open field test. GluK3 KO mice traveled significantly shorter distances during the 10 min of test after risperidone injection. DMSO, n = 10; Ris, n = 10; *t*(18) = 3.477, *p* = 0.0027. (**g**) Percentage of time spent in the central area of the open field. The percentage of time in the center was significantly decreased in GluK3 KO mice administrated with risperidone (*t*(18) = 2.563, *p* = 0.0196). (**h**) Representative traces of risperidone-treated WT and GluK3 KO mice in the open field test. Data are mean ± SEM. ns, not significant; *p < 0.05, **p < 0.01, ***p < 0.001, versus DMSO: unpaired *t*-test.

Risperidone, an atypical antipsychotic drug, is known for its antagonistic activity towards D2R and 5-HT2AR^53^. A previous report has shown that the administration of risperidone in mice reduces learned-fear but induces innate-fear responses, suggesting that risperidone may act as an anxiety inducer^54^. Therefore, we investigated whether risperidone-induced anxiety is intact in GluK3 KO mice. To examine this, we conducted the elevated plus maze test 30 min after intraperitoneal injection of risperidone (0.04 mg kg^−1^) or 0.1% DMSO. In WT mice, we found a significant decrease in the time spent in open arm (*t*(20) = 3.045, *p* = 0.0064, Fig. 6a) and increased time spent in the closed arm (*t*(20) = 3.028, *p* = 0.0066, Fig. 6a) following administration of risperidone, suggesting the expression of anxiety in WT mice. Importantly, GluK3 KO mice also showed a significantly increased time spent in the closed arm (*t*(20) = 3.045, *p* = 0.0064, Fig. 6b) and a reduced time in the open arm (*t*(18) = 1.866, *p* = 0.0784, Fig. 6b), suggesting that risperidone affects the anxiety-like behavior on both WT and GluK3 KO mice when examined by the elevated plus maze test (Fig. 6c). To further confirm the spontaneous motor activity and the anxiety-like behavior induced by risperidone, we performed the open field test. We found that risperidone significantly suppresses the motor activity of WT and GluK3 KO mice (WT: *t*(22) = 4.609 , *p* = 0.0001; GluK3 KO: *t*(18) = 3.477, *p* = 0.0027; Fig. 6d,f). An increased time spent in the center area of GluK3 KO mice, a sign of anxiolytic-like behavior, was reduced by risperidone administration to the levels of risperidone-injected WT mice (*t*(18) = 2.563, *p* = 0.0196; Fig. 6g). Although, we did not observe reduced center time in risperidone-injected WT mice (Fig. 6e), the moving traces of both genotypes that received risperidone displayed some noticeable dark spots, indicating that mice stopped their movement during the test (Fig. 6h). Indeed, we found that both WT and GluK3 KO mice spent significantly longer time at the corner of the maze after risperidone administration (Fig. S3).

**Figure 7:**
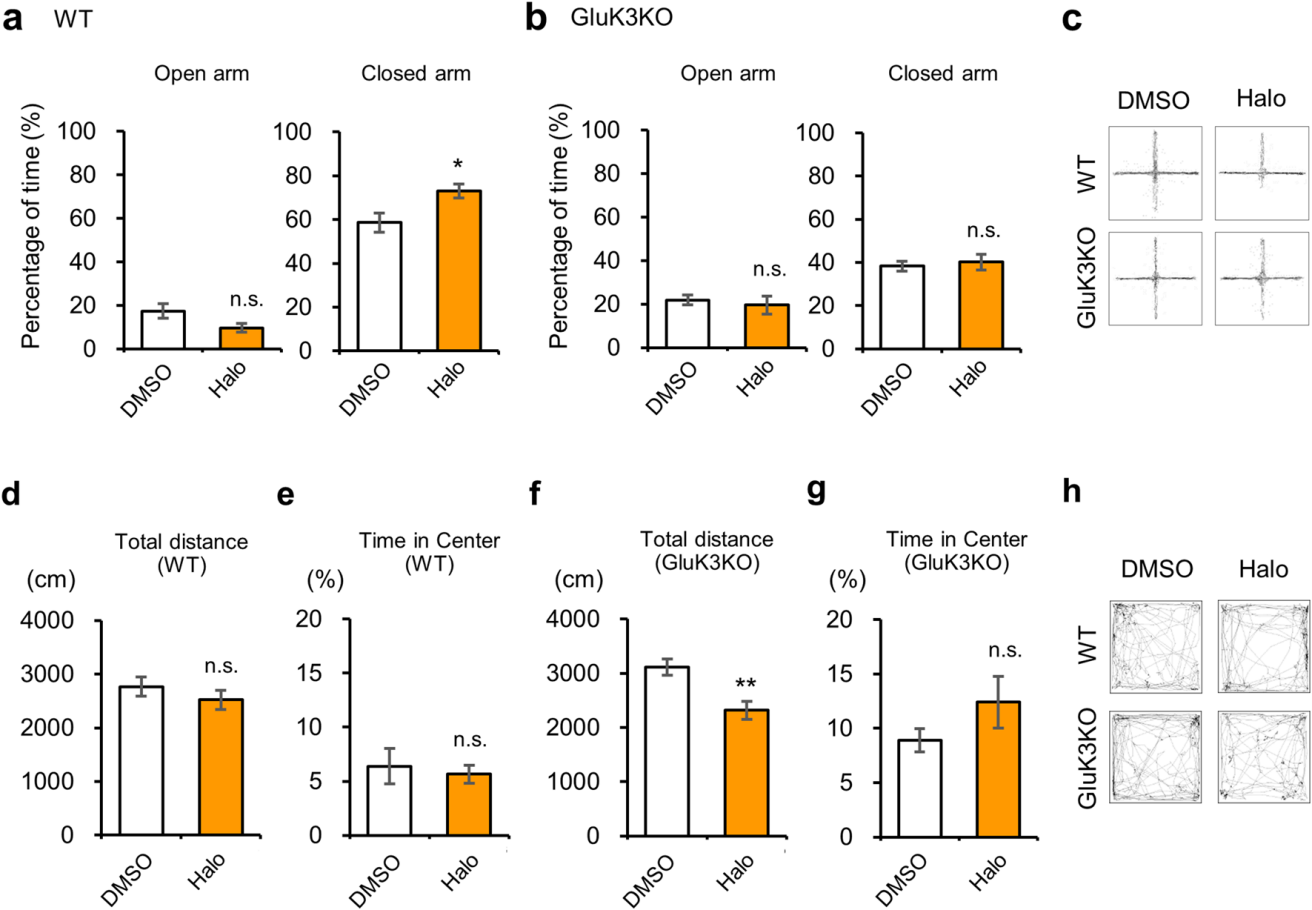
Effects of haloperidol on anxiety-like behavior in WT and GluK3 KO mice. (**a**) Percentage of time spent in both open and closed arms in WT mice treated with DMSO or haloperidol (Halo; 0.1 mg kg-1) during the 10 min of elevated plus maze test (DMSO: n = 12; Halo: n = 15; Open arm: *t*(25) = 1.973, *p* = 0.0596; Closed arm: *t*(25) = 2.601, *p* = 0.0154). (**b**) Percentage of time spent in both open and closed arms in GluK3 KO mice. Haloperidol did not induce anxiety-like behavior in GluK3 KO mice (DMSO, n = 18, Halo, n = 16: Open arm: *t*(32) = 0.4895, *p* = 0.6278; Closed arm: *t*(32) = 0.4352, *p* = 0.6664). (**c**) Representative traces of haloperidol-treated WT and GluK3KO mice in the elevated plus maze test examined for 10 min. (**d**) Total distance travelled in the open field in WT mice. No change in distance traveled during the 10 min of test was observed in WT mice after haloperidol injection. DMSO, n = 12; Halo, n = 13; *t*(23) = 0.9572 , *p* = 0.3484. (**e**) Percentage of time spent in the central area of the open field in WT mice. No difference was observed in WT mice injected with haloperidol. WT: *t*(23) = 0.4063 , *p* = 0.6883. (**f**) Total distance travelled in GluK3 KO mice DMSO, n = 18; Halo, n = 16; *t*(32) = 3.3558, *p* = 0.0012. (**g**) Percentage of time spent in the central area of the open field in GluK3 KO mice. No difference was observed in GluK3 KO mice injected with haloperidol. GluK3: *t*(32) = 1.393, *p* = 0.1732. (**h**) Representative traces of haloperidol-treated WT and GluK3KO mice in the open field test for 10 min. Data are presented as mean ± SEM. ns: not significant; *p < 0.05, **p < 0.01 versus DMSO: unpaired *t*-test.

5-HT2AR is the major target of risperidone, however, risperidone also acts on D2R and other molecules with lower affinity. To identify whether the effect of risperidone found in GluK3 KO mice was due to the antagonization of 5-HT2AR or D2R, we next tested the anxiety-like behavior using different antipsychotic drug haloperidol, a selective antagonist for D2R^53,55^. We performed the elevated plus maze test 1 h after intraperitoneal injection of haloperidol (0.1 mg kg^−1^) or 0.04% DMSO (Fig. 7a–c). We observed a reduced time in the open arm in haloperidol-administrated WT mice than the DMSO-administrated mice; however, this difference was not statistically significant (*t*(25) = 1.973, *p* = 0.0598, Fig. 7a). Contrary to the open arm, we observed a significant increase in the time spent in the closed arm in haloperidol-administrated WT mice (*t*(25) = 2.601, *p* = 0.015, Fig. 7a), suggesting the expression of anxiety-like behavior by haloperidol. However, haloperidol did not affect the behavioral phenotypes in GluK3 KO mice (open arm; *t*(32) = 0.4895, *p* = 0.6278, closed arm *t*(32) = 0.4352, *p* = 0.6664, (Fig. 7b). The total distance traveled in two genotypes in the elevated plus maze tests was reduced by haloperidol administration, suggesting that the effect of haloperidol on locomotor activity was intact in GluK3 KO mice (Fig. S4a,b). In contrast to the elevated plus maze test, the total distance traveled examined by the open field test (Fig. 7h) was unchanged in haloperidol-injected WT mice but it was significantly reduced in haloperidol-administered GluK3 KO mice (WT: *t*(23) = 0.9572, *p* = 0.3484; GluK3 KO: *t*(32) = 3.3558, *p* = 0.0012, Fig. 7d,f). The time spent in the center area of each genotype was unaltered by the administration of haloperidol compared with the DMSO-injected groups (Fig. 7e,g). These results suggested that haloperidol specifically induce anxiety-like behavior to WT in elevated plus maze test.

### The expression of D2R in the striatum is decreased in GluK3 KO mice

**Figure 8:**
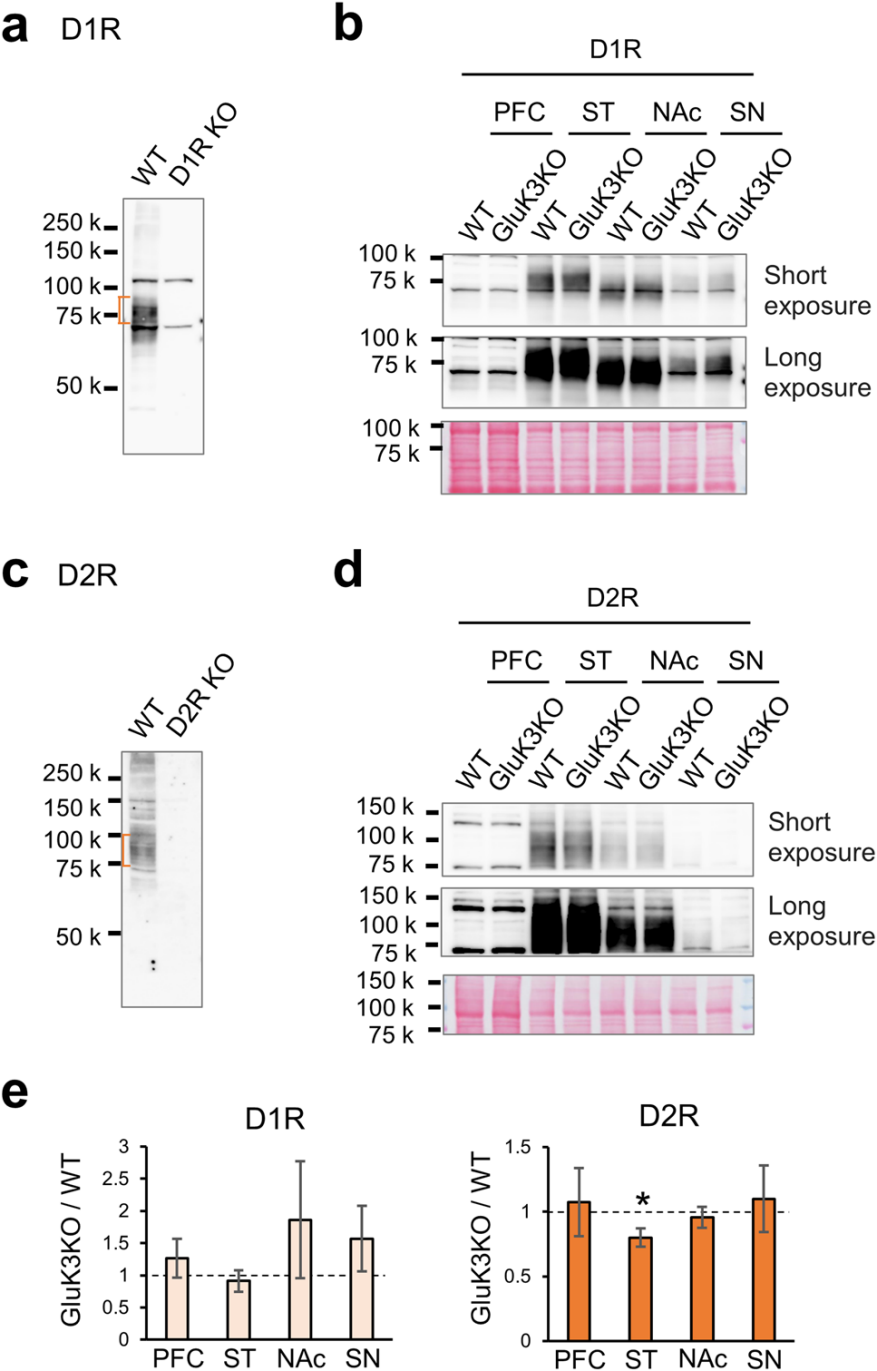
Effects of GluK3 deficiency on dopamine receptor D1R and D2R expression. Specificity of D1R (**a**) and D2R (**c**) antibodies was examined by western blotting using striatal P2 fraction prepared from WT, D1R KO, and D2R KO mice brain. Equal amounts of samples (50 μg) were loaded in each lane. Bands marked in orange are the expected size of receptors. (**b**,**d**) Representative blots for D1R (**b**) and D2R (**d**) in prefrontal cortex (PFC), striatum (ST), nucleus accumbens (NAc), and substantia nigra (SN) prepared from WT and GluK3 KO mice. Equal amounts of samples were loaded in each lane (PFC: 40 μg, ST: 20 μg, NAc: 24 μg, SN: 20 μg). D1R and D2R were normalized using Ponceau S (bottom of each blot) as a loading control. Short exposure and long exposure are shown. (**e**) Graphs showing that the expression of D1R **(**left) in all GluK3 KO brain regions is similar to WT brain, but significantly lower expression for D2R (right) is seen in the striatum of GluK3 KO brain (*p* = 0.0286). Data are mean ± SEM. n = 4. ns, not significant; *p < 0.05: Mann–Whitney U test.

Since we found that haloperidol, a selective antagonist for D2R, does not induce anxiety in GluK3 KO mice, we measured the expression levels of dopamine receptors in the brain. To confirm the band of D1R and D2R in western blotting, we used striatum extracts from WT mice and D1R- and D2R-deficient mice, as previously described^45^. The D1R antibody recognized receptors around 75 kDa in WT mice and this was ablated in D1R KO mice (Fig. 8a). Likewise, the D2R antibody recognized receptor subunits around 90 kDa (Fig. 8c). We then quantified the levels of D1R and D2R in the PFC, striatum, nucleus accumbens, and substantia nigra prepared from WT and GluK3 KO mice (Fig. 8b,d). We did not identify changes in the expression of D1R in GluK3 KO (Fig. 8e). However, the expression of D2R in the GluK3 KO striatum was significantly decreased compared to WT (*t*(6) = 2.2853, *p* = 0.032, n = 4; Fig. 8e). These results suggested that GluK3 signaling may be associated with the expression of D2R in the striatum.

## Discussion

**Figure 9:**
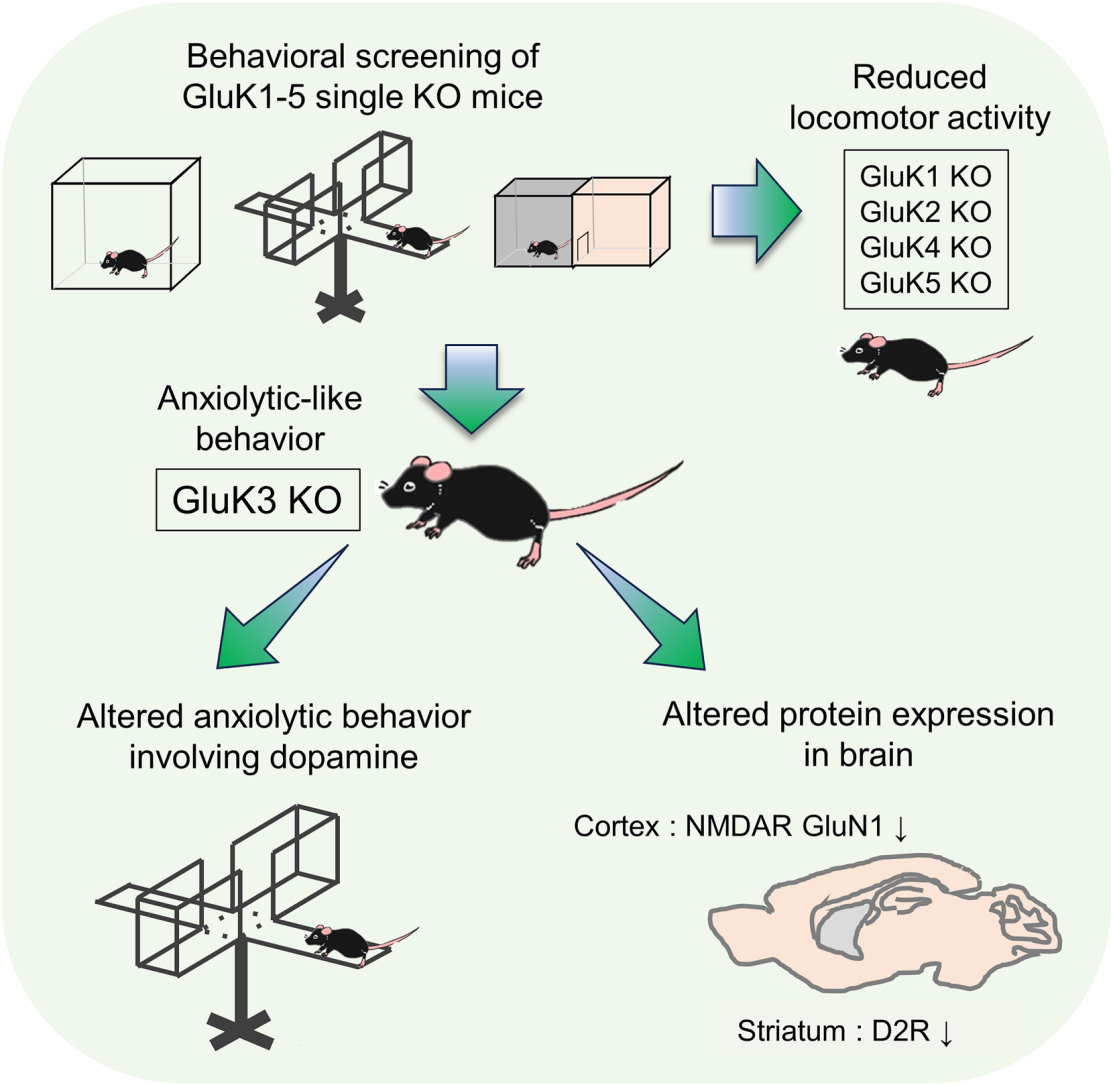
Summary of research flow and the findings.

KARs have been known for their multifunction in regulating neuronal activity^32,56,57^. There is a number of studies showing the association between KARs and psychiatric disorders^30^ and several KAR subunit deficient mice have displayed some of these phenotypes. However, the difference in the genetic background have been found to affect behavioral phenotypes in multiple mouse models of human diseases^36,42^, thus we generated all five KAR subunit (GluK1-5) KO mice in a pure C57BL/6N background and performed behavioral studies (Table 1). In this study, we demonstrated for the first time that the ablation of GluK3 leads to an anxiolytic-like behavior in mice, and GluK3 subunits in the cortex do not couple with high-affinity GluK4 and GluK5 subunits. Additionally, we found that the dopamine D2R antagonist haloperidol-induced anxiety maybe altered in GluK3 KO mice. These findings propose the role of GluK3-containing low-affinity KAR in anxiety behavior and their possible link to D2R-mediated signaling pathways (Fig. 9).

**Table 1:** Summary of behavioral phenotypes in GluK1-5 KO mice from current study.

Test	Open field		Elevated plus maze	Light and dark	Three-chamber	Tail suspension	Learning and memory
	Locomotor activity	Anxiety	Anxiety	Anxiety	Sociability	Depression	Context/cue
GluK1 KO	↓	–	↑	↑	N/A	N/A	N/A
GluK2 KO	↓	–	↓	–	N/A	N/A	N/A
GluK3 KO	–	↓	↓	–	–	↓(partial)	–
GluK4 KO	↓	–	–	–	N/A	N/A	N/A
GluK5 KO	↓	–	–	–	N/A	N/A	N/A

↑ increase, ↓ decrease, – not determined, N/A not assessed.

In our studies, we found that GluK3 is an important molecule for regulating anxiety, but not for basal locomotor activity, sociability, and conditioned memory. The role of GluK3 in anxiety is supported by the fact that the amount of GluK3 expressed in the CNS reflects the level of anxiety-related behavior. From immunoprecipitation assay, we identified that GluK3 couple with low-affinity GluK2 but not with high-affinity GluK4/GluK5, suggesting that GluK3-containing KARs are low-affinity KARs at least in the mouse cortex. Function of GluK2/3-KARs has been reported from the study that hippocampal mossy fiber synaptic plasticity is regulated by the presynaptic KARs composed of low-affinity GluK2 and GluK3^38,58^. Deletion of GluK2 subunits express a trend toward anxiolytic-like behavior in the elevated plus maze test, which is similar to that in GluK3 KO mice. However, GluK2 KO mice also display other phenotypes, such as altered spontaneous activity^35^. In addition, we have previously reported that GluK2 KO mice exhibit enhanced depressive-like behavior and reduced motivation toward their environment^36^, which we did not observe in GluK3 KO mice. This maybe because GluK2 subunit has diverse functions attributed to its ability to interact with all five GluK subunits^18,52,59^.

In this study, we conducted behavioral tests using mice with the same genetic background and were able to identify abnormal behaviors, as well as distinct patterns of behavior for each subunit. For example, we identified anxiety-like behavior and lowered locomotor activity in GluK1 KO mice, which matched with the previous reports using different genetic background^33,60^. The phenotypes of GluK2 KO mice in open field and elevated plus maze tests was rather similar to those of GluK3 KO mice but not GluK1 KO mice. Our study supports that GluK2/GluK3-containing low-affinity KARs contribute to the expression of anxiolytic behavior. Although, we cannot exclude the possibility that GluK1/GluK3-containing low-affinity KARs is present in the cortex, due to lack of specific antibody against GluK1.

Immunohistochemistry using specific GluK3 antibody, ensured the localization of GluK3 protein in the mouse brain. Unexpectedly, the distribution of GluK3 subunits were relatively weak in the central, lateral, and basolateral amygdala, one of the key regions known for controlling anxiety and emotion^61,62^, while GluK3 was enriched in the cortex including mPFC, ACC, and DEn. The mPFC is known for its association with anxiety, since coordinated activity of amygdala-mPFC circuits induced by threatening stimuli leads to the expression of anxiety-like behavior^63^. The neuronal activity in the ACC and DEn has also been demonstrated for their positive correlation with anxiety as measured using the elevated plus maze^64,65^. Therefore, our findings suggest that the GluK3-containing neural circuits in these brain regions might be responsible for regulating anxiety-related behavior. Future studies including genetical labeling of GluK3-positive neural circuits and the specific activation of these circuits will provide insights into the responsible brain regions for GluK3 that control anxiety-like behavior.

In our pharmacological analysis, we observed that the anxiety-like behavior mediated by D2R was impaired in GluK3 KO mice at least in the elevated plus maze test, while 5-HT2AR-induced anxiety was intact. Additionally, the expression level of D2R in the GluK3 KO mouse striatum was reduced compared to that in WT brain. There are shreds of evidence that dopamine plays a prominent role in modulating anxiety-related behaviors. For instance, the injection of the D2R antagonist in the amygdala, hippocampus, NAc, and VTA lead to both anxiogenic- and anxiolytic-like behavior in rat, which indicates their complicated regulation of anxiety through multiple brain regions^66^. Furthermore, a complete loss of KAR subunits in mice resulted in decreased spine density of spiny projection neurons (SPNs) in the striatum as well as deficits in corticostriatal input to SPNs including reduced NMDA:AMPA amplitude ratio, reduced field potential response and lower mEPSC frequencies^67^. Thus, the striatum could be the candidate region for anxiety that is controlled by GluK3-containing KARs. Further functional studies are required to identify how GluK3 signaling alter the expression of D2R in the striatum and if striatal D2Rs involve in the anxiolytic effects of GluK3 KO mice.

Previous studies have reported the anxiolytic activity of NMDAR glycine site antagonists^68^. GluN1 subunit mutant mice carrying point mutations in the glycine binding site GluN1-D481N have also exhibited an impairment in LTP, spatial learning and anxiety related behaviors^69^. In our western blotting, the expression of GluN1 protein was specifically decreased in the GluK3 KO mouse cortex, providing a possible association of GluK3 with NMDAR in the anxiety behavior. Future studies are required if the ablation of GluK3 derivatively collapse excitatory neurotransmission in the CNS through NMDARs.

In the present study, we demonstrated the function of GluK3 by performing animal behavior in KO mice and examined the subunit composition and brain localization of GluK3 using our subunit specific antibody. The expression of GluK3 was prominent in the cortex. Notably, GluK3 KO mice showed robust anxiolytic-like behavior, together with decreased NMDAR expression in the cortex and D2R in the striatum. Dopamine D2R antagonist haloperidol failed to induce anxiety-like behavior in GluK3 KO mice in the elevated plus maze test, therefore there may be a physiological link between GluK3 and D2R. A major limitation of our study is the difficulty in identifying the region- and cell-specific functions of GluK3. To find a precise role of GluK3-expressing neurons in the cortex, striatum, and any brain regions related to anxiety, genetic approaches that allow selective targeting of anxiety-related neural circuits such as glutamatergic, GABAergic, and dopaminergic neurons is required, while sparing neighboring GluK3-expressing cells.

### Supplementary Information

Supplementary Figures.

Supplementary Figure 5.

## Data Availability

The data that support the findings of this study are available from the corresponding author, M.T., upon reasonable request.
