# Supplementary material for: Behavioral analysis of kainate receptor KO mice and the role of GluK3 subunit in anxiety
Source: Sci Rep. 2024 Feb 24;14:4521. doi: 10.1038/s41598-024-55063-z (PMC10894277; doi:10.1038/s41598-024-55063-z)
Supplement: Supplementary file 1 — Supplementary Figures. [file 41598_2024_55063_MOESM1_ESM.docx]

**Supplementary Information**

**Behavioral analysis of kainate receptor KO mice and the role of GluK3 subunit in anxiety**

Izumi Iida, Kohtarou Konno, Rie Natsume, Manabu Abe, Masahiko Watanabe, Kenji Sakimura, Miho Terunuma

Supplementary Figure S1

**Figure S1. Behavioral analysis of five GluK subunit KO mice**

**a-d** Open Field test. **a** Distance traveled per movement. Reduced distance/movement was seen in GluK KO mice except for GluK3 KO mice. **b** Moving speed. Reduced moving speed was found in GluK KO mice except for GluK3 KO mice. **c** Locomotion frequency. Reduced moving speed was observed in GluK2 KO mice. **d** Total walking distance of WT littermate mice. No significant difference was observed in WT produced from GluK1-GluK5 KO mouse line. **e** Number of arm entry in an elevated plus maze test. Altered arm entry was seen in GluK1 KO, GluK2 KO and GluK3 KO mice. **f-g** Light/dark transition test. **f** Transition number. Reduced transition number was observed in GluK1 KO, GluK2 KO, and GluK5 KO mice. **g** Total walking distance. Reduced total distance traveled was seen in GluK1, GluK2 KO mice and increased total distance traveled was seen in GluK3 KO mice. Data are mean ± SEM. **p* < 0.05, ***p* < 0.01, ****p* < 0.001, *****p* < 0.0001 versus WT mice: one-way ANOVA with Dunnett’s multiple comparisons test.

Supplementary Figure S2

**Figure S2. Analysis of sociability and social novelty preference in three-chamber social interaction test**

**a** Sociability test. No difference in the preference to enter the social zone of mouse 1 was observed between WT and GluK3 KO mice (sociability index; *t*(37) = 1.142, *p* = 0.2609). **b** Social novelty preference test. No difference in the preference to enter the social zone of mouse 2 was observed between WT and GluK3 KO mice (social novelty index; *t*(36) = 0.616, *p* = 0.5418). **c** Percentage of time spent in each chamber (box) in sociability test. No change was observed between genotypes. **d** Percentage of time spent in each chamber in social novelty preference test. No change was observed between genotypes. **e** Number of entries in each chamber (box) in sociability test. No change was observed between genotypes. **f** Number of entries in each chamber (box) in social novelty preference test. No change was observed between genotypes. Data are mean ± SEM. ns, not significant; versus WT mice: unpaired *t*-test.

Supplementary Figure S3

**Figure S3. Effects of risperidone on anxiety-related behavior in WT and GluK3 KO mice**

**a** Time spent at the corner of maze in WT mice treated with DMSO or risperidone (Ris) (0.04 mg kg-1) during the 10 min of open field test (DMSO, n = 10; Ris, n = 13; *t*(21) = 2.519, *p* = 0.02). **b** Time spent at the corner of maze in GluK3 KO mice treated with DMSO or risperidone during the 10 min of open field test (DMSO, n = 10; Ris, n = 9; *t*(17) = 3.525, *p* = 0.033). Both WT and GluK3 KO mice spent longer time at the corner of the maze after risperidone administration. Data are mean ± SEM. **p* < 0.05, versus DMSO: unpaired *t*-test.

Supplementary Figure S4

**Figure S４. Effects of haloperidol on anxiety-like behavior in WT and GluK3 KO mice**

**a** Total distance travelled in the elevated plus maze test during the 10 minutes of test in WT mice. DMSO, n = 12; Halo, n = 15; *t*(25) = 2.661 , *p* = 0.0134. **b** Total distance travelled in the elevated plus maze test in GluK3 KO mice. DMSO, n = 18; Halo, n = 16; *t*(32) = 3.171 , *p* = 0.0033. Total distance traveled was significantly reduced in both WT and GluK3 KO mice after haloperidol injection. Data are mean ± SEM. **p* < 0.05, ***p* < 0.01, versus DMSO: unpaired *t*-test.
