# Supplementary material for: Behavioral analysis of kainate receptor KO mice and the role of GluK3 subunit in anxiety
Source: Sci Rep. 2024 Feb 24;14:4521. doi: 10.1038/s41598-024-55063-z (PMC10894277; doi:10.1038/s41598-024-55063-z)

Supplementary Figure S4

Uncropped western blotting images (left panels) and overlay of membrane picture including pre-stained marker with chemiluminescence (right panels)

Fig. 2a GluK3

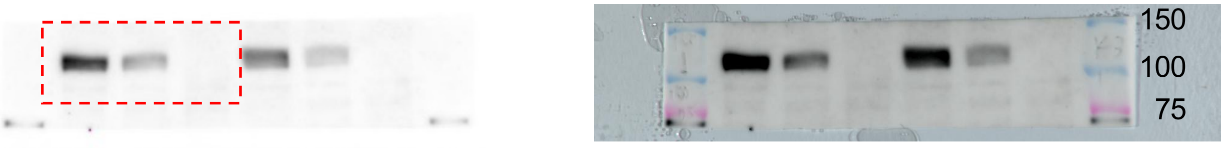

Fig. 5a GluK2

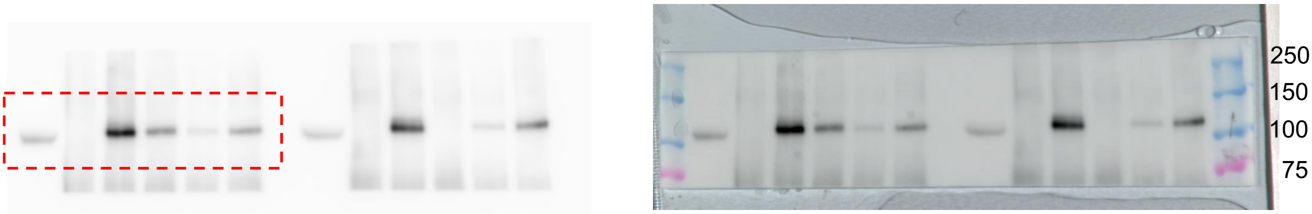

Fig. 5a GluK3

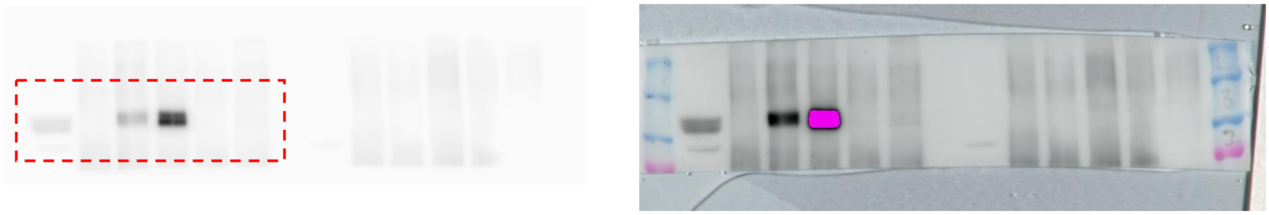

Fig. 5a GluK4

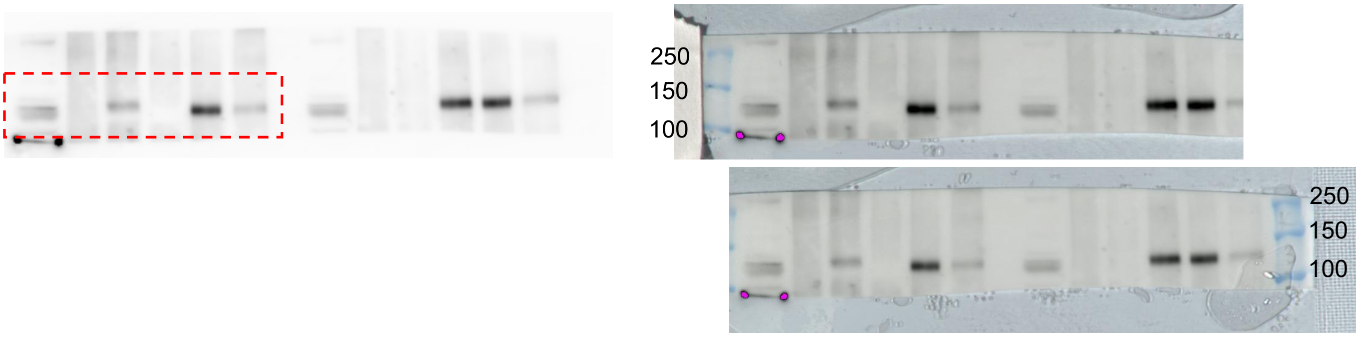

Fig. 5a GluK5

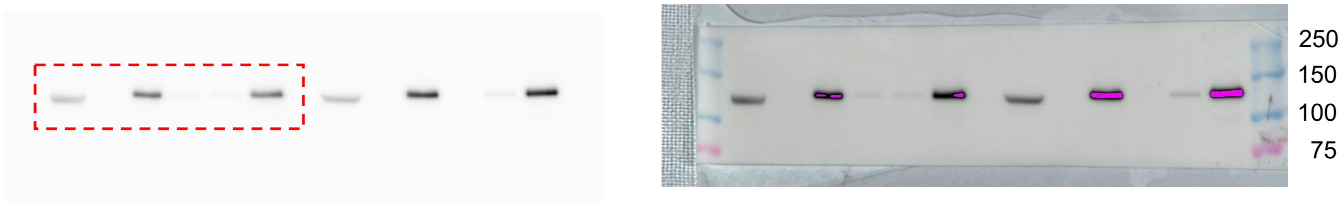

Fig. 5b GluK2

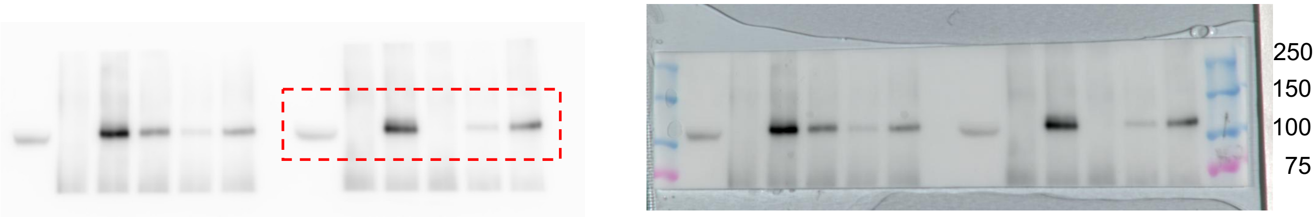

Fig. 5b GluK3

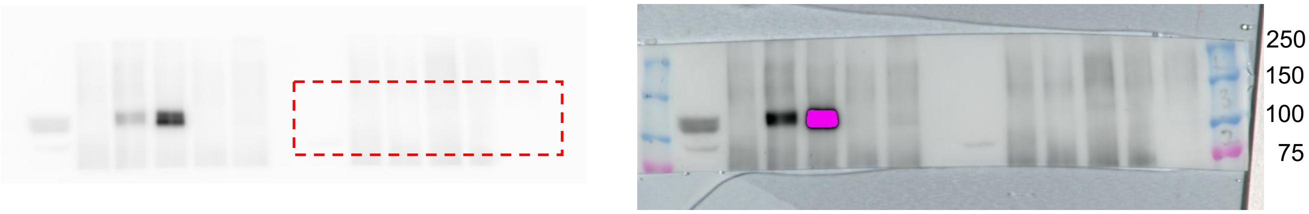

Fig. 5b GluK4

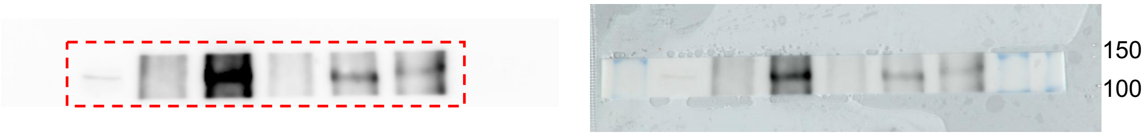

Fig. 5b GluK5

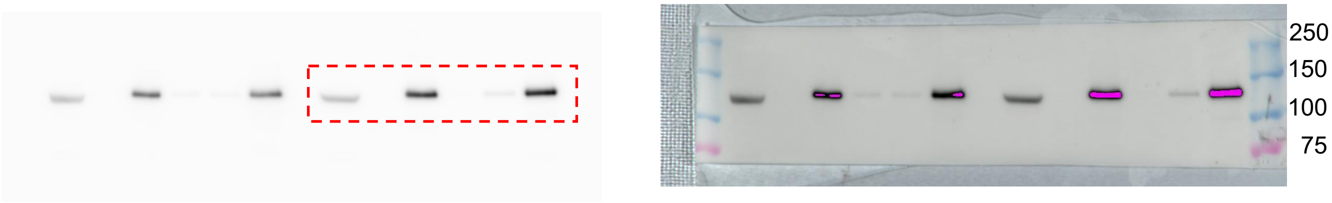

Fig. 5c GluN1

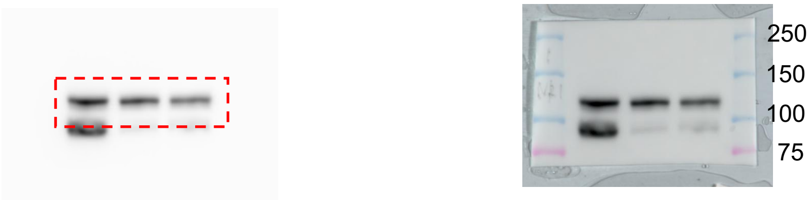

Fig. 5c GluN2A

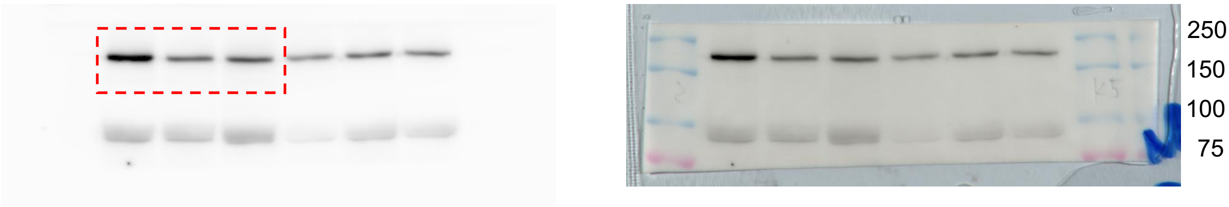

Fig. 5c GluN2B

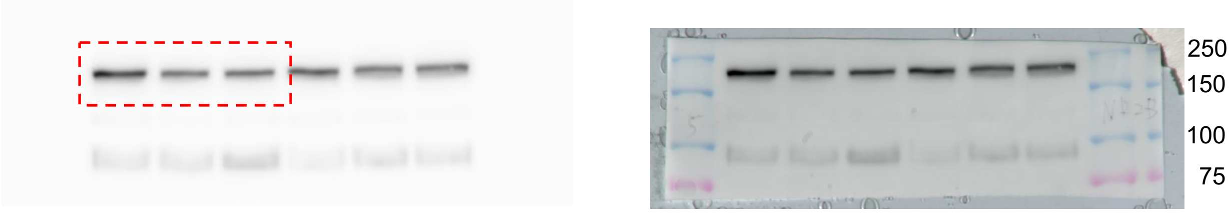

Fig. 5c GluA1

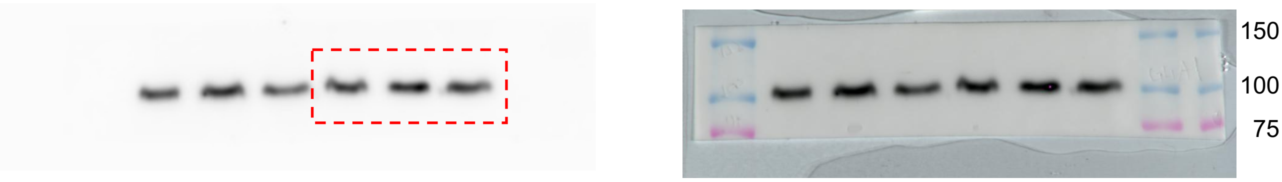

Fig. 5c GluA2

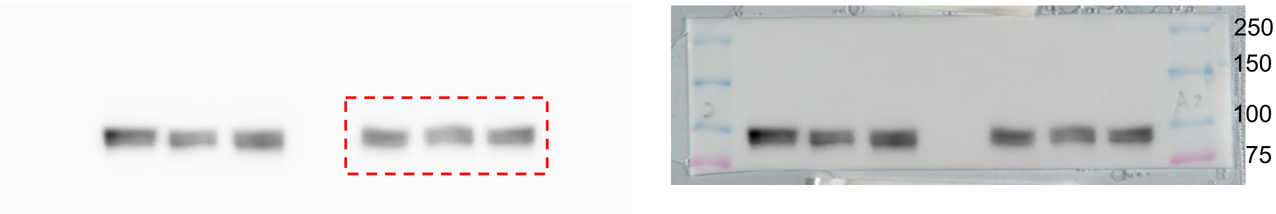

Fig. 5c GluA3

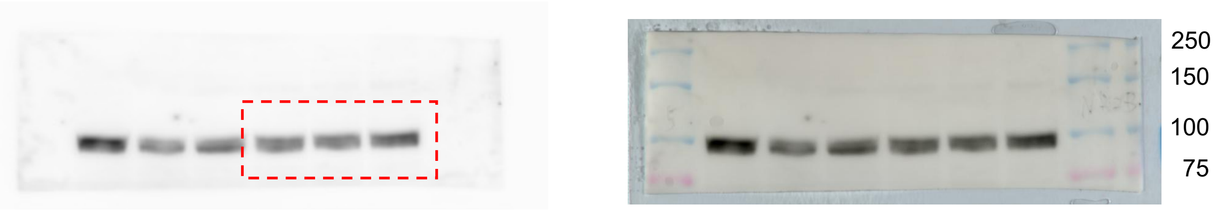

Fig. 5c GluK2

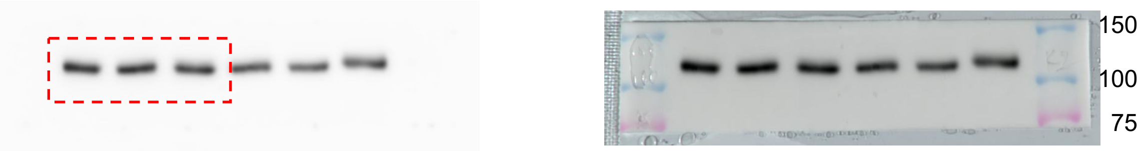

Fig. 5c GluK5

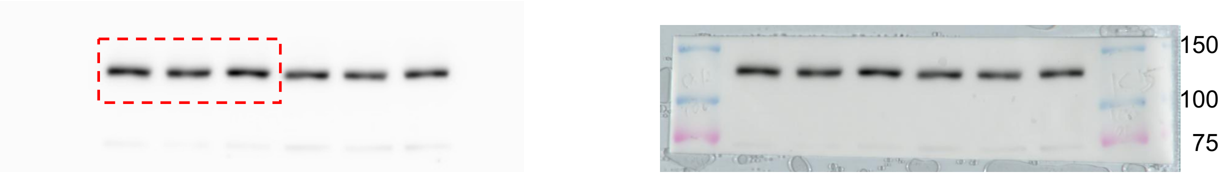

Fig. 8a D1R

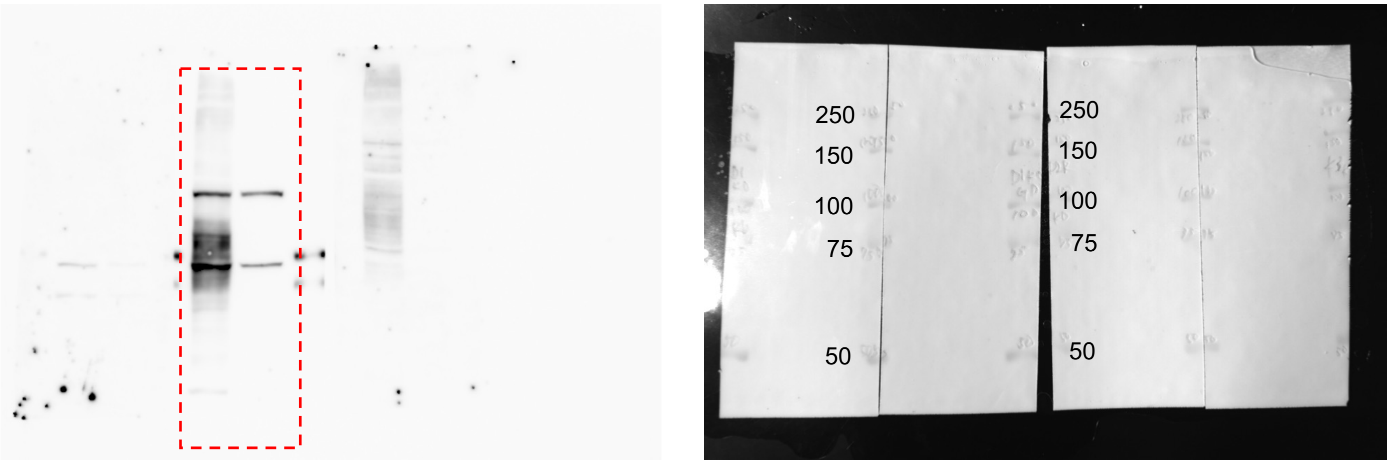

Fig. 8b D1R

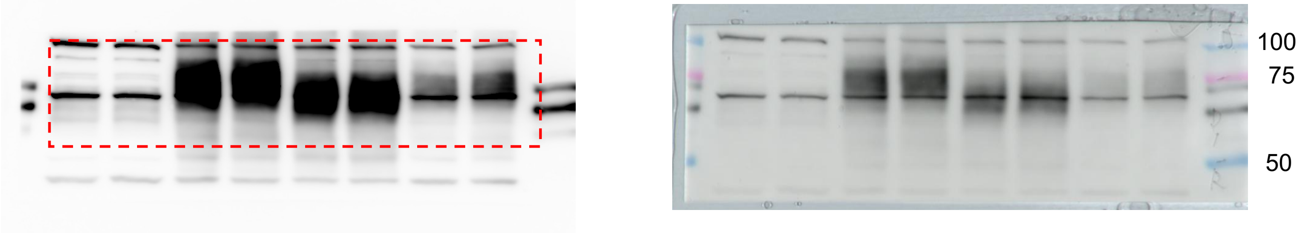

Fig. 8c D2R

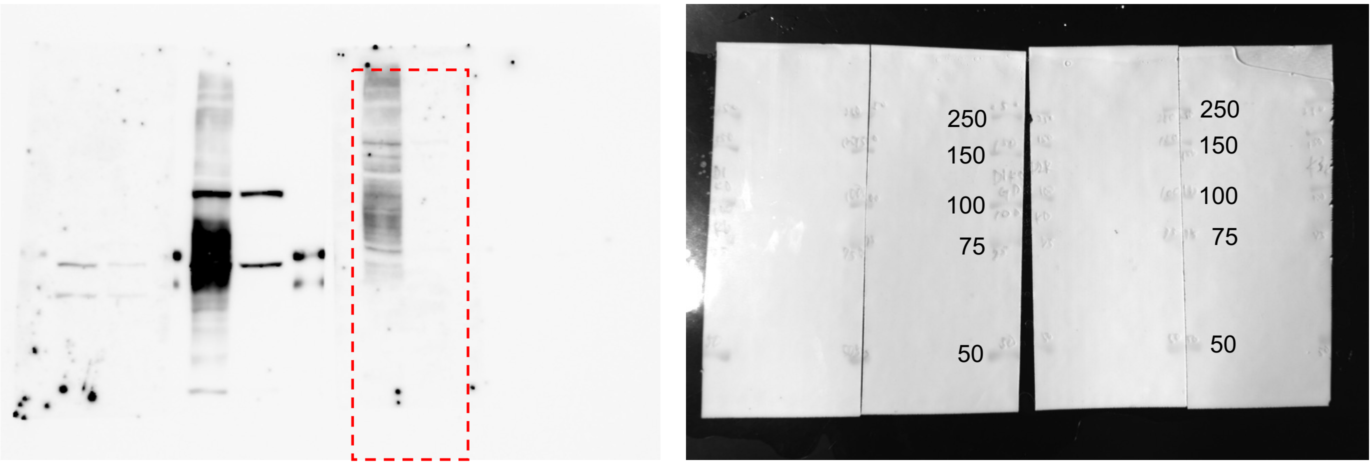

Fig. 8d D2R

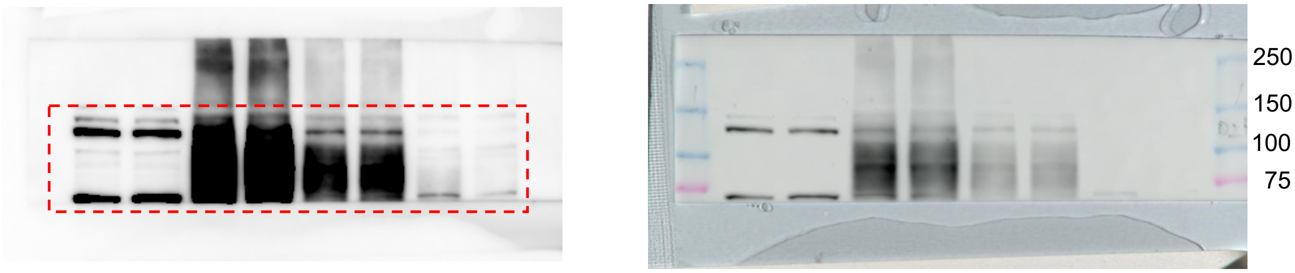

Supplement: Supplementary file 2 — Supplementary Figure 5. [file 41598_2024_55063_MOESM2_ESM.pdf]
